# Structural investigation of pathogenic *RFC1* AAGGG pentanucleotide repeats reveals a role of G-quadruplex in dysregulated gene expression in CANVAS

**DOI:** 10.1093/nar/gkae032

**Published:** 2024-01-24

**Authors:** Yang Wang, Junyan Wang, Zhenzhen Yan, Jianing Hou, Liqi Wan, Yingquan Yang, Yu Liu, Jie Yi, Pei Guo, Da Han

**Affiliations:** School of Materials Science and Engineering, Tianjin University, Tianjin 300350, China; Zhejiang Cancer Hospital, Hangzhou Institute of Medicine (HIM), Chinese Academy of Sciences, Hangzhou, Zhejiang 310022, China; Zhejiang Cancer Hospital, Hangzhou Institute of Medicine (HIM), Chinese Academy of Sciences, Hangzhou, Zhejiang 310022, China; School of Biology and Biological Engineering, South China University of Technology, Guangzhou, Guangdong 510006, China; Institute of Molecular Medicine (IMM) Renji Hospital, School of Medicine, Shanghai Jiao Tong University, Shanghai 200127, China; Zhejiang Cancer Hospital, Hangzhou Institute of Medicine (HIM), Chinese Academy of Sciences, Hangzhou, Zhejiang 310022, China; School of Biology and Biological Engineering, South China University of Technology, Guangzhou, Guangdong 510006, China; Zhejiang Cancer Hospital, Hangzhou Institute of Medicine (HIM), Chinese Academy of Sciences, Hangzhou, Zhejiang 310022, China; Zhejiang Cancer Hospital, Hangzhou Institute of Medicine (HIM), Chinese Academy of Sciences, Hangzhou, Zhejiang 310022, China; Zhejiang Cancer Hospital, Hangzhou Institute of Medicine (HIM), Chinese Academy of Sciences, Hangzhou, Zhejiang 310022, China; Zhejiang Cancer Hospital, Hangzhou Institute of Medicine (HIM), Chinese Academy of Sciences, Hangzhou, Zhejiang 310022, China; Institute of Molecular Medicine (IMM) Renji Hospital, School of Medicine, Shanghai Jiao Tong University, Shanghai 200127, China

## Abstract

An expansion of AAGGG pentanucleotide repeats in the replication factor C subunit 1 (*RFC1*) gene is the genetic cause of cerebellar ataxia, neuropathy, and vestibular areflexia syndrome (CANVAS), and it also links to several other neurodegenerative diseases including the Parkinson's disease. However, the pathogenic mechanism of *RFC1* AAGGG repeat expansion remains enigmatic. Here, we report that the pathogenic *RFC1* AAGGG repeats form DNA and RNA parallel G-quadruplex (G4) structures that play a role in impairing biological processes. We determine the first high-resolution nuclear magnetic resonance (NMR) structure of a bimolecular parallel G4 formed by d(AAGGG)_2_AA and reveal how AAGGG repeats fold into a higher-order structure composed of three G-tetrad layers, and further demonstrate the formation of intramolecular G4s in longer DNA and RNA repeats. The pathogenic AAGGG repeats, but not the nonpathogenic AAAAG repeats, form G4 structures to stall DNA replication and reduce gene expression via impairing the translation process in a repeat-length-dependent manner. Our results provide an unprecedented structural basis for understanding the pathogenic mechanism of AAGGG repeat expansion associated with CANVAS. In addition, the high-resolution structures resolved in this study will facilitate rational design of small-molecule ligands and helicases targeting G4s formed by AAGGG repeats for therapeutic interventions.

## Graphical Abstract

**Graphical Abstract:**
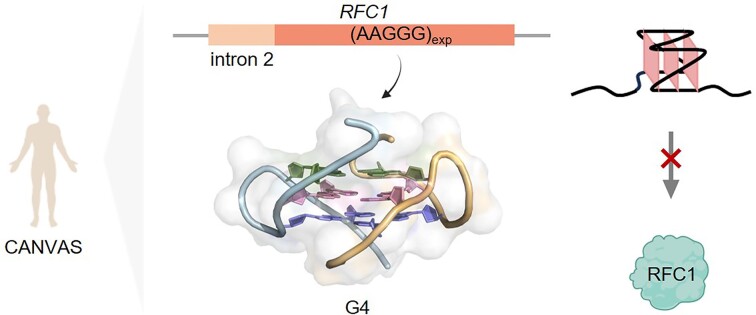

## Introduction

**Figure 1.**
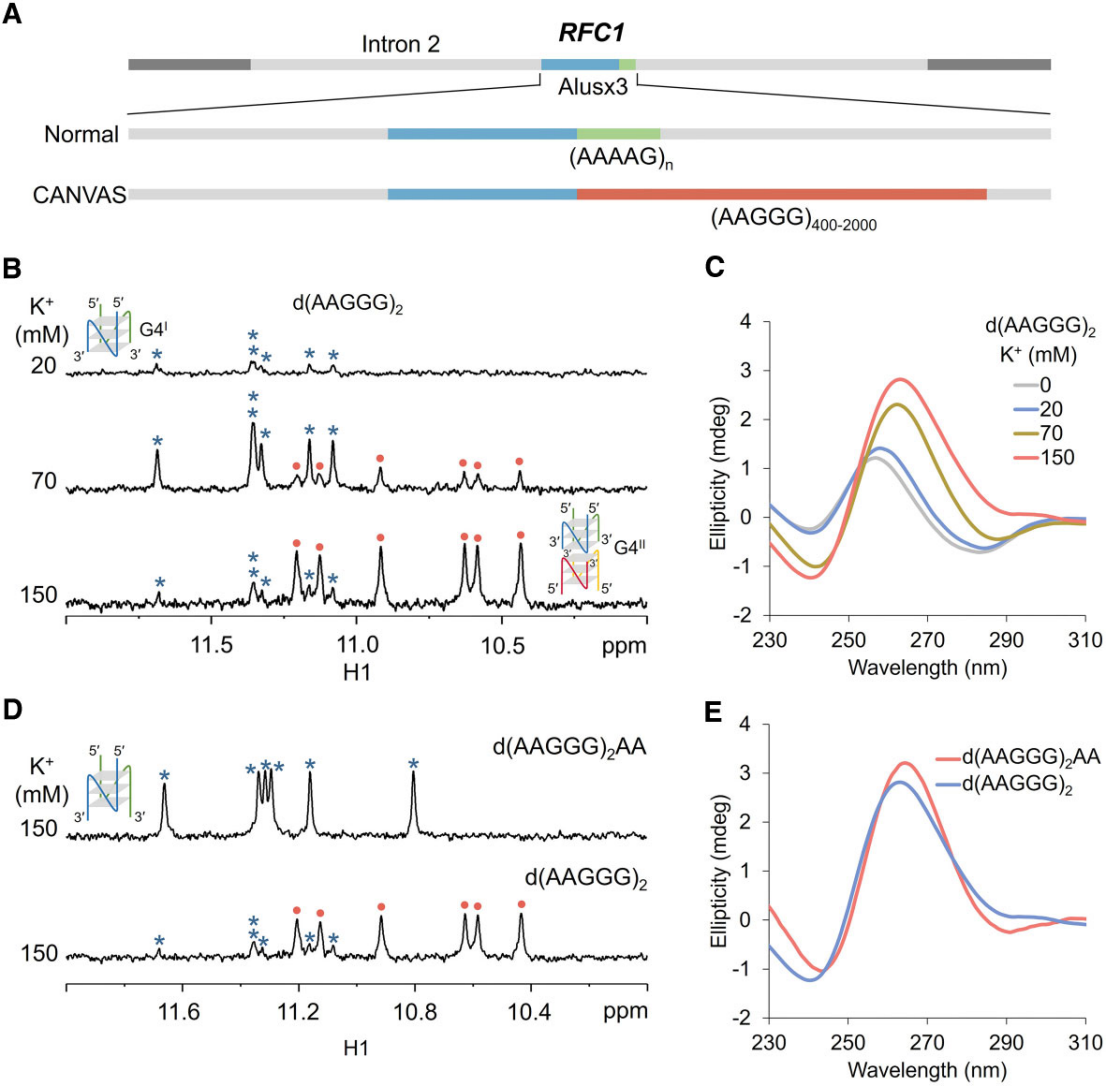
(**A**) Schematic shows the nonpathogenic AAAAG repeats in normal *RFC1* gene and AAGGG repeat expansion in pathogenic *RFC1* gene of CANVAS patients. (**B**, **C**) 1D ^1^H NMR (imino proton region) and CD spectra of d(AAGGG)_2_ at variable K^+^ concentrations, with blue asterisks and red dots indicating NMR signals from the bimolecular G4^I^ and tetramolecular G4^II^, respectively. (**D**, **E**) 1D ^1^H NMR and CD spectra of d(AAGGG)_2_AA in 150 mM K^+^. The ^1^H NMR and CD spectra of d(AAGGG)_2_ are equivalent to those shown in (B, C). [DNA] = 100 μM for NMR and 20 μM for CD, [NaPi, pH 7] = 1 mM, [KCl] = 0/20/70/150 mM, 25°C.

Nucleotide repeat expansion diseases constitute some of the most common inherited neurodegenerative diseases, including the Huntington's disease (HD), amyotrophic lateral sclerosis (ALS) and frontotemporal dementia (FTD) (1–3). Recently, an expansion of AAGGG pentanucleotide repeats in intron 2 of the replication factor C subunit 1 (*RFC1*) gene has been identified as the genetic cause of cerebellar ataxia, neuropathy, and vestibular areflexia syndrome (CANVAS), a neurological disorder of autosomal recessive inheritance (4–7). The normal *RFC1* contains a benign adenine-rich (AAAAG)_n_ or (AAAGG)_n_ configuration in the polyA tail of an Alu element, with the former reported as the most common nonpathogenic allele (6). In contrast, at least five different expanded repeat motifs have been observed in the pathogenic *RFC1* allele with the guanine-rich (AAGGG)_400–2000_ having the highest frequency in CANVAS patients (Figure 1A). *RFC1* is a vital gene encoding the large subunit of replication factor C, which is an essential DNA polymerase accessory protein required for DNA replication and repair in human cells (8). Recently, it has been reported that the *RFC1* AAGGG repeat expansion causes reduced levels of full-length RFC1 protein in CANVAS patients (9,10), but the underlying molecular mechanism remains elusive. Besides, increasing evidence has also suggested a linkage between the *RFC1* AAGGG repeat expansion and several other neurodegenerative diseases including the Parkinson's disease (PD) (11,12), multiple system atrophy (MSA) (13–15) and chronic idiopathic axonal polyneuropathy (CIAP) (16,17). These together raise an importance and urgency to tease out the structure and function of the pathogenic *RFC1* AAGGG repeats.

Noncanonical nucleic acid structures have been widely recognized as pathogenic drivers and drug targets in human diseases including cancers and neurodegenerative diseases (18–23). G-quadruplex (G4) structures formed by guanine-rich sequences are known to be key players in various biological processes associated with normal physiology and disease pathology, especially with their roles in gene regulation most well characterized (24,25). High-throughput sequencing has identified more than 700000 putative G4 sites in the human genome (26), among which some G4 sites are long tracts of nucleotide repeats concatenated to neurodegenerative diseases (27,28). For instance, the DNA and RNA G4 structures formed by expanded GGGGCC repeats from *C9orf72* gene are found to participate in aberrant molecular cascades, including DNA replication, transcription and translation, which lead to the development of ALS-FTD neurodegenerative diseases (29–31). Recently, Abdi *et al.* have reported that AAGGG repeats formed G4 structures *in vitro* primarily based on circular dichroism (CD) spectroscopic analysis (32). However, the high-resolution structures of AAGGG repeats, which can shed light on ligand design and pathogenic mechanism study, remain unavailable.

To provide a high-resolution structural basis for understanding how pathogenic *RFC1* AAGGG repeats fold into higher-order structures and how they affect key biological processes such as the dysregulated gene expression, here we performed a solution nuclear magnetic resonance (NMR) spectroscopic investigation to characterize the structural features of AAGGG repeats, followed by structure-based molecular and cellular biology assays to elucidate the effects of G4 formation on several key biological processes. We determined the high-resolution NMR structures of a bimolecular parallel DNA G4 formed by (AAGGG)_2_AA in K^+^ solution, and further demonstrated that intramolecular G4s could form in longer DNA and RNA AAGGG repeats. By utilizing the small-molecule ligand N-methyl mesoporphyrin IX (NMM) that bound and stabilized the G4 structures of AAGGG repeats, we showcased that G4 formation in AAGGG repeats could impede DNA polymerase processivity to cause replication stalling *in vitro*, and reduce gene expression via impairing the translation but not transcription process in cells in a repeat-length-dependent manner. In contrast, the nonpathogenic AAAAG repeats did not affect DNA replication and gene expression. Our results for the first time report the G4 structure as a fundamental determinant for the pathogenic *RFC1* AAGGG repeats linked to gene dysregulation, providing a possible molecular mechanism for the pathogenesis of relevant neurodegenerative diseases.

## Materials and methods

### Sample preparation

DNA and RNA oligonucleotides were purchased from Sangon Biotech Co. Ltd (Shanghai, China) and Accurate Biology (Hunan, China), respectively, with high-performance liquid chromatography (HPLC) purification. The purchased DNA and RNA were further purified in our laboratory using diethylaminoethyl sephacel anion exchange column and centrifugal desalting. The 6% site-specific ^15^N isotopically labeled DNA oligonucleotides were synthesized on a K&A H8 synthesizer using the 2′-deoxyguanosine phosphoramidite (98% ^15^N) purchased from Cambridge Isotope Laboratories (USA), and purified by HPLC, diethylaminoethyl sephacel anion exchange column and centrifugal desalting. DNA and RNA samples were quantified using a NanoDrop microvolume spectrophotometer.

### NMR spectroscopy

NMR samples contained 100 μM DNA (for one-dimensional, 1D experiments) or 500 to 800 μM DNA (for two-dimensional, 2D experiments), 1 mM NaPi (pH 7), 0 to 150 mM KCl, 90% H_2_O/10% D_2_O or 99.96% D_2_O, and 0.02 mM sodium 4,4-dimethyl-4-silapentane-1-sulfonate (DSS) as the internal reference. The newly prepared NMR samples were stored at room temperature for overnight prior to NMR spectrum acquisition. Excitation sculpting and pre-saturation water suppression methods were applied for 90% H_2_O/10% D_2_O and 99.96% D_2_O samples, respectively. 2D NMR experiments, including nuclear Overhauser effect spectroscopy (NOESY) (mixing times of 100 to 300 ms), double quantum-filtered correlation spectroscopy (DQF-COSY), total correlation spectroscopy (TOCSY) (mixing time of 75 ms), ^1^H-^13^C heteronuclear single quantum correlation (HSQC) (^1^*J*_C-H_ of 180 Hz), ^1^H-^15^N HSQC (^1^*J*_N-H_ of 90 Hz) and ^1^H−^13^C heteronuclear multiple bond coherence (HMBC) with an evolution period of 65 ms were acquired at 25°C. The H8 and H1′ resonances were assigned from NOESY, and the sugar proton (H2′, H2″, H3′, H4′, H5′ and H5″) resonances were assigned from DQF-COSY and/or TOCSY spectra. The guanine H1 resonances were unambiguously assigned from ^1^H−^15^N HSQC spectra using the site-specific 6% ^15^N-labeled DNA samples. The adenine H2 resonances were assigned using ^1^H−^13^C HMBC. NMR spectra were acquired on a Bruker AVANCE 600 MHz spectrometer and analyzed using TopSpin3.6.2 software.

### Structural calculations

For structural calculations of d(AAGGG)_2_AA, NMR-derived distance restraints were obtained by integrating NOE cross peaks from NOESY spectra. NOE-derived distance restraints were classified as strong (1.8–4.0 Å), strong or medium (2.5–4.5 Å), medium (3.0–5.0 Å), medium or weak (3.5–5.5 Å) and weak (4.0–6.0 Å) according to NOE intensity. A wider range of 1.8–6.0 Å was applied for seriously overlapped NOEs. Glycosidic torsion angles of 90–330° for *anti* were applied based on intranucleotide H8–H1′ NOE intensity and purine C8 chemical shifts. The H1′−C1′−C2′−H2′ dihedral angles were determined by ^3^*J*_H1′-H2′_ coupling constants measured from the DQF-COSY and Karplus equation (33). The structural calculations were performed as previously described (34,35) on AMBER18 (36) using the bsc1 force field (37). The starting models were energy minimized, and then subjected to restrained molecular dynamic (rMD) simulations and restrained energy minimization (rEM). Briefly, the system temperature was increased from 300 to 600 K at the first 5 ps, maintained at 600 K for 20 ps, decreased to 300 K within 5 ps, and stayed at 300 K for 5 ps during the rMD process. The structural coordinates were then subjected to rEM by 200 steps of the steepest descent and conjugated gradient minimization steps until the energy gradient difference between successive minimization steps was smaller than 0.1 kcal mol^−1^Å^−2^. Among the 500 structures calculated with random seeds, 10 structures with the lowest total energies were selected as the final representative ensemble. The root-mean-square deviation (RMSD) values were calculated using the *suppose* module of AMBER. The 3D structures were prepared using PyMOL.

### Native polyacrylamide gel electrophoresis (PAGE)

DNA loading samples were prepared to contain 0.1 mM DNA, 1 mM NaPi (pH 7) and variable concentrations of K^+^ as stated in the figure legends, and then stored at room temperature for overnight. Native PAGE experiments were performed using 10−20% polyacrylamide gels supplemented with 1 × TBE buffer at room temperature. DNA bands were visualized by staining the gels with stains-all solutions.

### CD spectroscopy

CD samples were prepared to contain 20 μM DNA or RNA, 1 mM NaPi (pH 7) and variable K^+^ concentrations as stated in the figure legends, and then stored at room temperature for overnight. CD spectra were recorded on a Chirascan V100 spectrometer using 1 mm path length quartz cuvette and 1 nm bandwidth at room temperature. The blank correction was made by subtracting the buffer spectrum. For CD melting experiments, the CD ellipticity at 264 nm was recorded from 15 to 95°C with a heating rate of 1°C/min. CD melting curves were constructed using CD ellipticity at 264 nm as a function of temperature, and fitted with a two-state transition model (38) to determine the melting temperature (*T*_m_) of DNA G4.

### Fluorescence experiments

The 1 mM DNA or RNA stock samples were prepared in 1 mM NaPi (pH 7) and 150 mM KCl, and stored at room temperature for overnight. The DNA or RNA stock was titrated into the ligand solution (1 μM NMM) in 1 mM NaPi (pH 7) and 150 mM KCl. The solution was mixed well and equilibrated for 5 min. The emission spectra were measured using a 10 mm path length cuvette with an excitation wavelength of 393 nm and a recorded range of 550–750 nm. Fluorescence experiments were performed on an Edinburgh instruments FLS1000 spectrometer at room temperature.

### *In vitro* DNA replicational assay

The mixture of template and 5′-Cy5-labeled primer (1:1 equivalent) was annealed by heating at 95°C for 5 min and cooling to room temperate for overnight. For studying the effect of NMM-stabilized G4 formation on polymerase processivity, NMM was added into the reaction mixture at various concentrations and incubated at room temperature for 2 h. Primer extension was performed for 1 h at 37°C in a 20 μl reaction buffer containing 50 μM primer-template, 1.25 mM dNTPs (New England Biolabs, NEB), 0.3 U/μl Klenow fragment (KF) (NEB), 5 mM NaCl, 1 mM Tris−HCl and 1 mM MgCl_2_. For studying the effect of K^+^-stabilized G4 formation on polymerase processivity, the reaction buffer contained 0 to 150 mM K^+^. The primer extension products were resolved using 10% denaturing PAGE and visualized by Cytiva Amersham ImageQuant 800. The fluorescent gel images were analyzed using ImageJ.

### Cell culture and transfection

HEK293T cells were cultured in DMEM (Gibco) supplemented with 10% (v/v) fetal bovine serum (FBS, Cytiva) at 37°C with 5% CO_2_. Transient transfection of the plasmid construct into HEK293T cells was conducted using Lipofectamine 3000 reagent (Thermo Fisher Scientific) according to the manufacturer's recommendations. In brief, 1000 ng plasmid, 1 μl P3000 in 25 μl Opti-MEM (Gibco), and 1 μl Lipofectamine 3000 in 25 μl Opti-MEM were mixed and incubated for 15 min at room temperature. The two pBudCE4.1 vector-based plasmid constructs were purchased from GenScript (Nanjing, China).

### Confocal microscopy

HEK293T cells were plated in eight-well microscope slide with 2 × 10^5^ cells/well and cultured for overnight. 4 h after the transfection, cells were cultured for 24 h in a fresh medium containing no ligand or 3 μM NMM with 0.1% DMSO, and then washed with PBS. A fresh Phenol Red-free DMEM media with 10% FBS was added prior to confocal experiment. The confocal microscopy was conducted on a Leica Stellaris 8 instrument. A 405 and 488 nm laser excitations were used to image BFP and EGFP, respectively.

### Flow cytometry

HEK293T cells were plated in a six-well plate with 5 × 10^5^ cells/well and cultured for overnight. 4 h after the transfection, cells were cultured for 24 h in a fresh medium containing no ligand, NMM (1, 3 μM) with 0.1% DMSO. Then the cells were washed with PBS and resuspended in fresh PBS with 4% FBS for flow cytometry experiment using a Thermo Fisher Attune NxT instrument.

### Reverse transcription and quantitative PCR (RT-qPCR)

RNA was isolated using RNAiso Plus (TAKARA), followed by reverse transcription using Prime Script RT reagent Kit with gDNA Eraser (TAKARA) according to the manufacture's protocol. Quantitative PCR was carried out with TB Green Premix Ex Taq (TAKARA) using Roche Light Cycler 96. Expression of EGFP was standardized using BFP as a reference, and relative levels of expression were quantified by calculating ${2}^{ - {\mathrm{\Delta \Delta }}{{\mathrm{C}}}_{\mathrm{T}}}$ where ${\mathrm{\Delta \Delta }}{{\mathrm{C}}}_{\mathrm{T}}$ is the difference in ${C}_{\mathrm{T}}$ (cycle threshold) between target and reference (39). The primer sequences are listed in Supplementary Table S1.

### Statistical analysis

Statistical analyses were performed using the GraphPad Prism 9.0. Data are given as means ± SD by three independent experiments. Quantitative analysis was performed using two-tailed Student's *t* test. *P* value < 0.05 was taken as statistically significant.

## Results

### Short d(AAGGG)_2_ and d(AAGGG)_2_AA formed intermolecular parallel G4 structures

We began with NMR investigation on the solution structure of a short sequence composed of two AAGGG repeats from the pathogenic *RFC1* gene (Figure 1A). In 20 mM K^+^, d(AAGGG)_2_ showed six guanine imino proton (G H1) signals at ∼11.0–11.6 ppm suggesting the formation of a first type of G4 structure, namely G4^I^. Further increasing the K^+^ concentration to 70/150 mM leads to the formation of a second type of G4 structure that exhibited six relatively upfield G H1 signals, namely G4^II^ (Figure 1B and Supplementary Figure S1). CD spectra of d(AAGGG)_2_ exhibited a maximum ellipticity at 264 nm and a minimum ellipticity at 240 nm in 70/150 mM K^+^ (Figure 1C), suggesting the formation of parallel G4 structures. It is noted that only six guanine residues could not form three G-tetrad layers intramolecularly, and thereby both G4^I^ and G4^II^ are intermolecular G4 structures. We further performed native PAGE for G4^I^ and G4^II^ using two reported reference G4s, i.e. the TAG that formed a three-layer bimolecular G4 (12 nt × 2) and the T30177 that formed a six-layer bimolecular G4 (17 nt × 2) (40,41), and revealed that G4^I^ and G4^II^ were bimolecular and tetramolecular structures, respectively (Supplementary Figure S2). We noted that the ratio of bimolecular and tetramolecular G4s shown in PAGE appeared to be different from that shown in NMR (Figure 1B), which was attributed to the absence of K^+^ ions in the gel and electrophoresis buffer. Plus, the more upfield G H1 signals of G4^II^ hinted that G4^II^ was likely to be a higher-order assembly of two G4^I^ through stacking between the 3′-terminal G-tetrads, as the 5′-flanking adenine residues would disrupt terminal stackings.

As targeting the G4 structure has become an emerging paradigm in therapeutic intervention for repeat expansion diseases (25,42), we then attempted to resolve the high-resolution structures of d(AAGGG)_2_ to guide ligand selection for further functional study. However, high-quality NMR spectra of d(AAGGG)_2_ were not available for a pure G4^I^ conformer neither in low K^+^ (co-existed with random coils) nor high K^+^ (co-existed with the tetramolecular G4^II^) (Figure 1B and Supplementary Figure S1). As G4^II^ was suspected to be an assemble of two G4^I^ structures by stacking at the 3′-termini, we then added one to two adenine residue(s) at the 3′-termini of d(AAGGG)_2_ to prevent 3′-terminal stackings and thus to eliminate the formation of tetramolecular G4. It should be noted that d(AAGGG)_2_A and d(AAGGG)_2_AA still preserve the repetitive sequence nature of the pathogenic *RFC1* AAGGG repeats. The native PAGE showed that d(AAGGG)_2_A still formed a tetramolecular G4 (Supplementary Figure S3), but encouragingly d(AAGGG)_2_AA formed a pure bimolecular parallel G4 that exhibited similar NMR and CD spectral features to the G4^I^ formed in d(AAGGG)_2_ (Figure 1D, E and Supplementary Figures S3–S5).

### Solution NMR structure of the bimolecular parallel G4 of d(AAGGG)_2_AA

**Figure 2.**
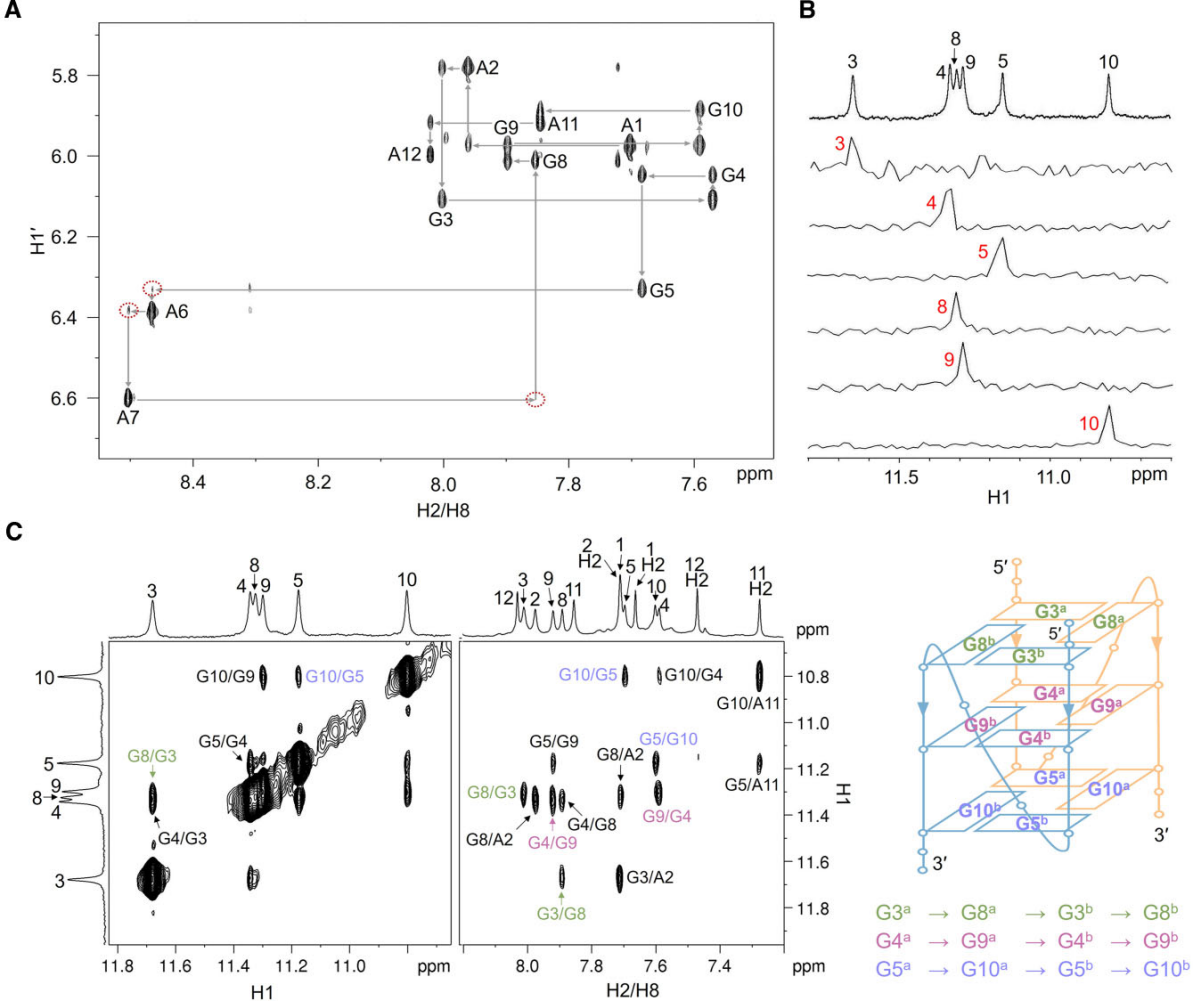
(**A**) The H6/H8-H1' fingerprint region from the NOESY spectrum of d(AAGGG)_2_AA. The weak sequential NOEs of loop residues are marked with red dotted circles. (**B**) ^15^N-filtered HSQC spectra of site-specific 6% ^15^N-labeled d(AAGGG)_2_AA for G H1 resonance assignment. (**C**) The H1–H1 and H8–H1 regions from the NOESY spectrum of d(AAGGG)_2_AA show NOEs connections in each G tetrad. The schematic of bimolecular parallel G4 of d(AAGGG)_2_AA wherein the two chains were shown in yellow and blue. [DNA] = 500 μM, [NaPi, pH 7] = 1 mM, [KCl] = 150 mM, 99.96% D_2_O and 25°C for (**A**), 10% D_2_O and 15°C for (**B**, **C**). Mixing time = 300 ms.

To determine the high-resolution structure of d(AAGGG)_2_AA, 2D NMR spectra, including NOESY, DQF-COSY, TOCSY, ^1^H–^13^C HSQC and ^1^H–^13^C HMBC, were acquired using unlabeled DNA sample to assign H8 and H1′ (Figure 2A), sugar protons and adenine H2 (Supplementary Figures S6–S10 and Supplementary Table S2). The guanine H1 resonances were unambiguously assigned through ^1^H-^15^N HSQC spectra using 6% ^15^N-labeled DNA sample at each designated guanine position (Figure 2B). Based on G H1-H8 NOE connections, the directions of Hoogsteen hydrogen bond donor-to-acceptor of three G-tetrads were determined to be G3^a^·G8^a^·G3^b^·G8^b^, G4^a^·G9^a^·G4^b^·G9^b^ and G5^a^·G10^a^·G5^b^·G10^b^, wherein the superscript a and b represent residues from two respective chains of the bimolecular G4, respectively (Figure 2C).

**Figure 3.**
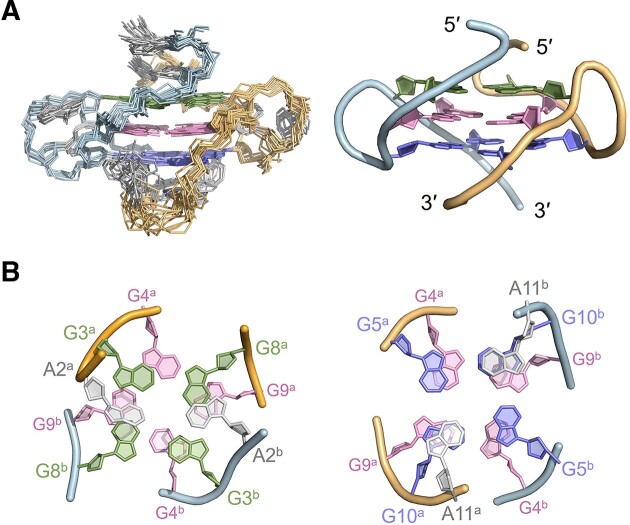
(**A**) Superimposed 10 representative solution NMR structures of d(AAGGG)_2_AA (PDB ID: 8X1V). For a clearer representation, one with the lowest total energy was chosen to show detailed structural features of the core. The G3^a^·G8^a^·G3^b^·G8^b^, G4^a^·G9^a^·G4^b^·G9^b^ and G5^a^·G10^a^·G5^b^·G10^b^ G-tetrads are shown in green, pink and blue, respectively. (**B**) The top view shows stackings between A2^a^/A2^b^, G3^a^·G8^a^·G3^b^·G8^b^ and G4^a^·G9^a^·G4^b^·G9^b^. The upward view shows stackings between A11^a^/A11^b^, G5^a^·G10^a^·G5^b^·G10^b^ and G4^a^·G9^a^·G4^b^·G9^b^.

The structures of d(AAGGG)_2_AA were calculated using rMD simulations with 248 NMR-derived distance restraints, 24 glycosidic torsion angle restraints, 4 sugar dihedral angle restraints, 48 hydrogen bond restraints and 36 G-tetrad planarity restraints, which are summarized in Supplementary Tables S3–S5. Among the 500 calculated structures with random seeds, ten structures with the lowest total energies were selected as the final representative ensemble (Figure 3A), and they were well converged with a heavy atom RMSD of 0.5 ± 0.1 and 1.0 ± 0.2 Å for the G-tetrad core and all residues, respectively (Table 1). Similar G4 structures could be obtained without using the G-tetrad planarity restraints during rMD calculations but exhibiting a relatively larger heavy atom RMSD than those calculated using G-tetrad planarity restraints (Supplementary Figure S11). The bimolecular parallel G4 of d(AAGGG)_2_AA contains three G-tetrads with every two layers exhibiting extensive base-base stackings (Figure 3B). More detailed structural features including the twist and rise between every two adjacent G-tetrads were analyzed using the WebTetrado tool (43) and shown in Supplementary Figure S12. To connect the three G-tetrad layers, the phosphodiester backbones of A6^a^/A6^b^ and A7^a^/A7^b^ were twisted, and this is consistent with the weak NOEs of G5 H1′-A6 H8, A6 H1′-A7 H8 and A7 H1′-G8 H8 (Figure 2A). A2^a^/A2^b^ stacked on the G3^a^·G8^a^·G3^b^·G8^b^ tetrad whereas A11^a^/A11^b^ stacked on the G5^a^·G10^a^·G5^b^·G10^b^ tetrad (Figure 3B), which agreed with NOEs of A2 H2-G3/G8 H1 and A11 H2-G5/G10 H1 (Figure 2C).

**Table 1.** NMR restraints and refinement statistics of d(AAGGG)_2_AA

**Structural restraints**	
NOE-derived distance	248
Glycosidic torsion angle (χ)	24
Sugar dihedral angle (H1′–C1′–C2′–H2′)	4
Hydrogen bond	48
G-tetrad planarity	36
**Details of restraint deviations**	
Number of distance deviation > 0.2 Å	0
Maximum distance deviation (Å)	0.19
Number of angle deviation > 5°	1
Maximum angle deviations (°)	5.4
**Deviations from ideal covalent geometry** **^a^**	
Bonds (Å)	0.0079 ± 0.0004
Angles (°)	2.41 ± 0.03
**Pairwise heavy atom RMSD (Å)** **^a^**	
All residues	1.0 ± 0.2
G-tetrad core	0.5 ± 0.1

^a^The mean and SD values were obtained from the 10 representative structures.

### Longer DNA and RNA AAGGG repeats formed intramolecular parallel G4s

**Figure 4.**
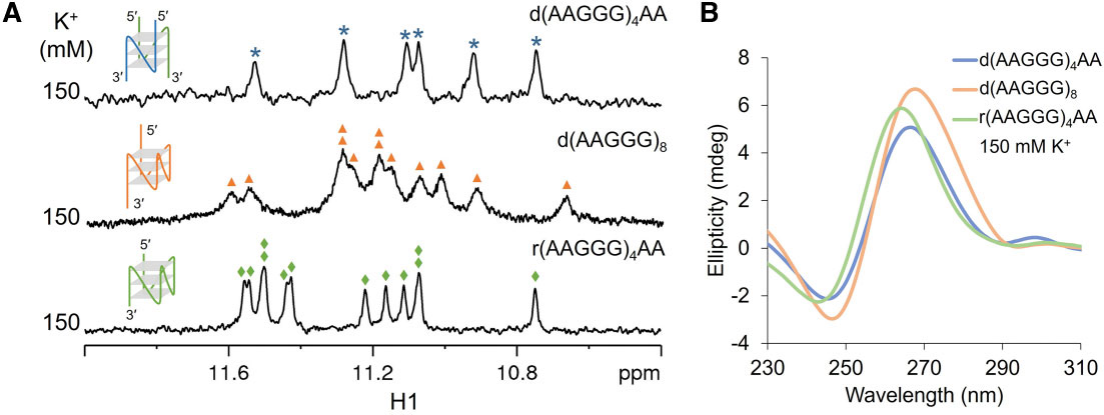
(**A**) 1D ^1^H NMR spectra of d(AAGGG)_4_AA, d(AAGGG)_8_ and r(AAGGG)_4_AA in 150 mM K^+^. Imino proton peaks of d(AAGGG)_4_AA, d(AAGGG)_8_ and r(AAGGG)_4_AA are labeled with blue asterisks, orange triangles and green diamonds, respectively. (**B**) CD spectra of d(AAGGG)_4_AA (blue), d(AAGGG)_8_ (orange) and r(AAGGG)_4_AA (green) in 150 mM K^+^. [DNA] = 100 μM for NMR and 20 μM for CD, [NaPi, pH 7] = 1 mM, [KCl] = 150 mM, 25°C.

Based on the 3D topology of the bimolecular parallel G4 of d(AAGGG)_2_AA (Figure 3), we expected that an intramolecular parallel G4 composed of three G-tetrad layers would be able to form when the number of AAGGG repeats equals or exceeds four. The 1D ^1^H NMR spectrum of d(AAGGG)_4_AA in 150 mM K^+^ showed six G H1 signals at ∼11.0 to 11.6 ppm similar to those of d(AAGGG)_2_AA (Figure 4A), suggesting that d(AAGGG)_4_AA possibly formed a bimolecular G4. The CD melting curves of d(AAGGG)_4_AA showed a higher *T*_m_ by ∼8°C at a DNA concentration of 20 than 5 μM, and the native PAGE further supported a bimolecular G4 state of d(AAGGG)_4_AA (Supplementary Figure S13). When the number of repeats increased, d(AAGGG)_8_ formed an intramolecular three-layer parallel G4 as suggested by twelve G H1 signals at ∼10.6–11.6 ppm and CD ellipticity at maximum of 264 nm and minimum of 245 nm (Figure 4A, B). The monomolecular G4 state of d(AAGGG)_8_ was further supported by similar *T*_m_ values at DNA concentrations of 20 and 5 μM, as well as the native PAGE result (Supplementary Figure S14).

In repeat expansion diseases, RNA transcripts harboring expanded repeats, e.g. r(CAG)_exp_ and r(GGGGCC)_exp_, form unusual structures that cause cellular dysfunctions such as abnormal translation and protein sequestration (31,44). The thermodynamic stability of RNA G4 is reported to be generally higher than that of DNA G4 (45), and therefore we speculated that RNA repeats may also form G4 structures to exert detrimental functions in CANVAS pathogenesis. We further performed NMR and CD experiments on r(AAGGG)_4_AA RNA. The twelve well-resolved G H1 signals at ∼10.8 to 11.6 ppm and CD ellipticity at maximum of 264 nm and minimum of 244 nm revealed the formation of an intramolecular parallel G4 (Figure 4A, B). Taken together, we have demonstrated that the pathogenic *RFC1* AAGGG repeats can form parallel G4 structures at both DNA and RNA molecular levels, with longer repeats tending to form intramolecular G4s. Although we have not assessed the structures of disease relevant sequence length (400–2000 repeats), the pathogenic *RFC1* gene harboring hundreds to thousands of AAGGG repeats is likely to form intramolecular G4s.

**Figure 5.**
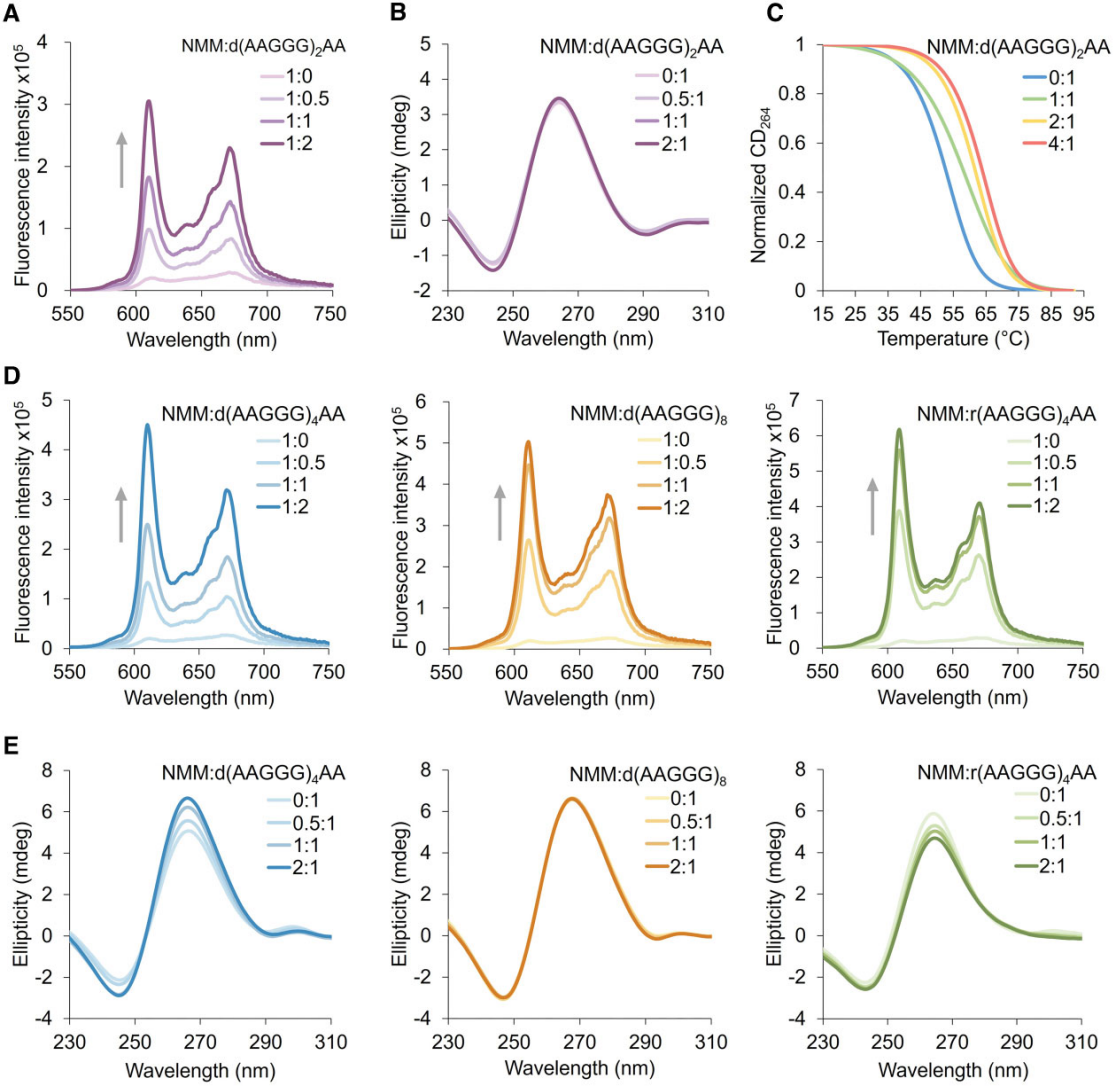
(**A**) Fluorescence spectra of NMM upon d(AAGGG)_2_AA titration. (**B**) CD spectra of d(AAGGG)_2_AA upon NMM titration. (**C**) CD melting curves of d(AAGGG)_2_AA at 0, 1, 2 and 4 equivalent(s) of NMM. The *T*_m_ of d(AAGGG)_2_AA was determined to be 52.3 ± 0.1, 58.2 ± 0.5, 61.8 ± 0.3 and 63.8 ± 0.1°C at 0, 1, 2 and 4 equivalent(s) of NMM, respectively. (**D**) Fluorescence spectra of NMM upon titrating d(AAGGG)_4_AA, d(AAGGG)_8_ and r(AAGGG)_4_AA to NMM. (**E**) CD spectra of d(AAGGG)_4_A, d(AAGGG)_8_ and r(AAGGG)_4_AA upon titrating NMM to DNA. [DNA] = 20 μM for CD. [NMM] = 1 μM for fluorescence experiments. [NaPi, pH 7] = 1 mM, [KCl] = 150 mM, 25°C.

After consolidating the formation of DNA and RNA intramolecular G4 structure in the pathogenic *RFC1* AAGGG repeats, we next sought to seek for a small-molecule ligand that can stabilize the G4 to assist our subsequent investigation on the pathogenic function of AAGGG repeats. By comparing the 3D structure of d(AAGGG)_2_AA with the structures of G4-ligand complexes available in the Protein Data Bank, the small-molecule ligand NMM emerges as an appropriate candidate as it binds to a parallel G4 with similar topology of our determined d(AAGGG)_2_AA (46). We then monitored the binding of NMM to d(AAGGG)_2_AA using NMR, fluorescence and CD spectroscopy. The 1D ^1^H NMR spectra of d(AAGGG)_2_AA showed a new set of upfield guanine H1 resonances at 0.5–2 equivalents of NMM (Supplementary Figure S15), suggesting the binding of NMM to d(AAGGG)_2_AA. However, NMR peak broadening of d(AAGGG)_2_AA occurred in the presence of NMM, which was possibly due to nonspecific bindings of NMM to d(AAGGG)_2_AA, e.g. binding at the 5′- and/or 3′-terminal G-tetrad. The binding interaction was further supported by significantly increased fluorescence at 610 nm of NMM upon adding pre-formed d(AAGGG)_2_AA G4 (Figure 5A). Besides, d(AAGGG)_2_AA retained its parallel G4 topology post binding with NMM as suggested by unaltered CD ellipticity at maximum of 264 nm and minimum of 244 nm (Figure 5B). The NMR, fluorescence and CD spectroscopic results collectively consolidate the binding of NMM to the G4 of d(AAGGG)_2_AA. In addition, the CD melting curves showed a significantly increased *T*_m_ of d(AAGGG)_2_AA by 6 to 11°C at 1 to 4 equivalents of NMM (Figure 5C). We further demonstrated the bindings of NMM to d(AAGGG)_4_AA, d(AAGGG)_8_ and r(AAGGG)_4_AA using fluorescence and CD spectra (Figure 5D, E). These lay an important foundation to tune the propensity of G4 formation and explore possible functions of G4 structures in pathogenic *RFC1* AAGGG repeats.

### G4 formation in pathogenic AAGGG repeats caused replication stalling *in vitro*

**Figure 6.**
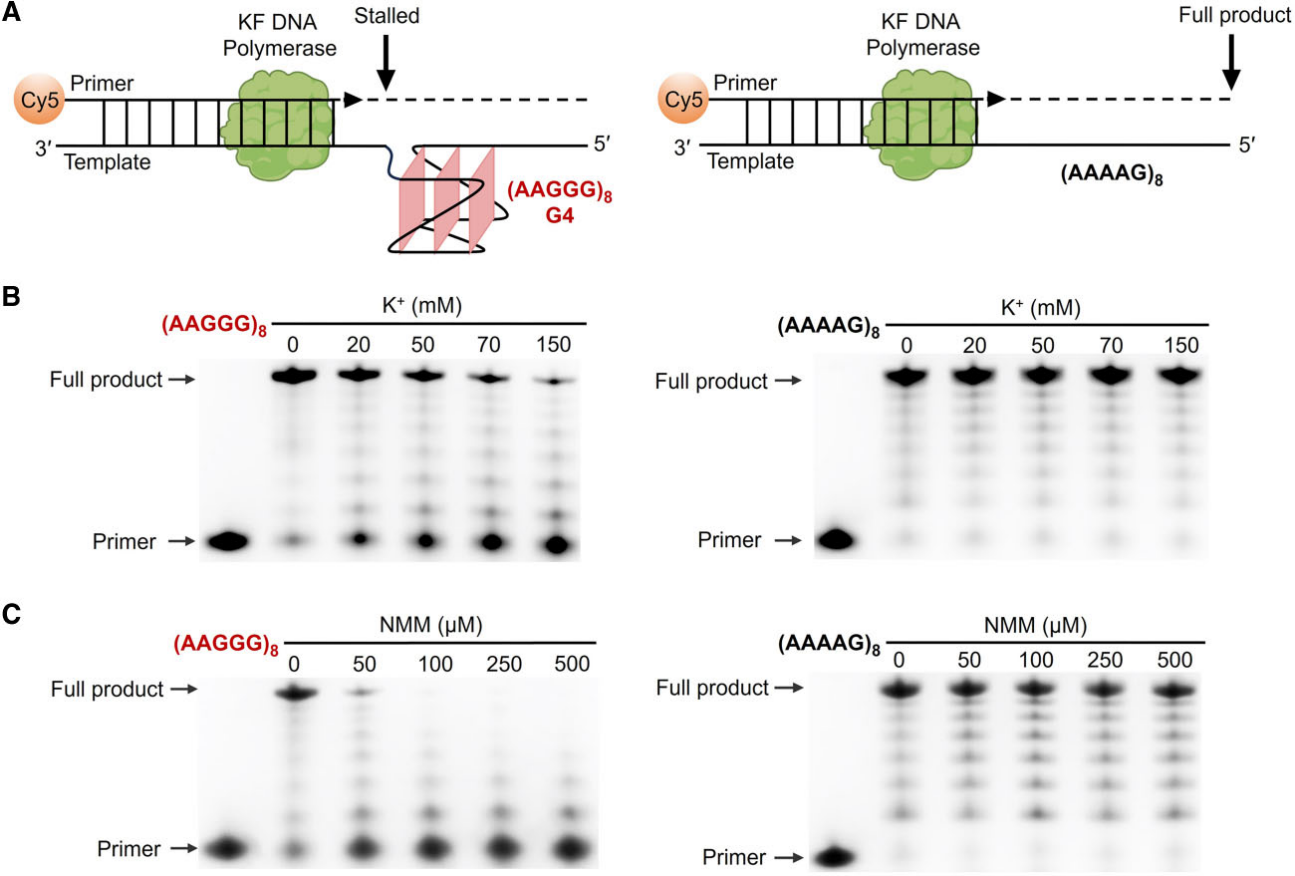
(**A**) Schematic of the *in vitro* KF extension assay on primer-template models containing pathogenic AAGGG repeats and non-pathogenic AAAAG repeats. (B, C) Denaturing PAGE results show KF extension products under various concentrations of K^+^ and NMM. The uncropped original gels for (**B**) and (**C**) are shown in Supplementary Figure S16, respectively. [K^+^] = 20 mM in (C).

Numbers of neurodegenerative diseases leading to ataxia and neuropathy are linked to DNA damage and abnormal repair pathways (47,48). Non-B DNA structures, in particular the G4s with high thermostability, can pose as obstacles for DNA polymerase processivity to cause replication stalling and DNA damage (49,50). Therefore, we first examined if the AAGGG repeats can form DNA G4 in an elongated template to impede polymerase processivity. For this aim, we established an *in vitro* replicational assay by placing eight AAGGG repeats, i.e. (AAGGG)_8_, in the template. Meanwhile, a reference template containing non-pathogenic (AAAAG)_8_ from normal *RFC1* allele was also prepared (Figure 6A). The 5′-Cy5-labeled primer was extended by the Klenow fragment (KF) of DNA polymerase I with 5′ to 3′ polymerase and 3′ to 5′ exonuclease activities. This KF was chosen as it has been commonly used to study effects of various types of non-B DNAs on polymerase processivity *in vitro* (34,51).

The *in vitro* replicational assays were first performed under variable K^+^ concentrations to assess the effect of G4 formation on polymerase processivity. As resolved by denaturing PAGE, the template containing (AAGGG)_8_ showed an efficient primer extension in the absence of K^+^. When the K^+^ concentration was increased, the population of fully extended products was gradually decreased and more non-extended primers were accumulated. Under a physiologically relevant K^+^ concentration of 150 mM, there was only a tiny population of fully extended products (Figure 6B, left). In contrast, the polymerase processivity was not affected by K^+^ concentration for the reference template containing (AAAAG)_8_, and the full-length products were efficiently synthesized (Figure 6B, right). We noted that the reference template containing (AAAAG)_8_ also generated tiny truncated replicational products, and this could be attributed to an intrinsic slowing down of DNA polymerase processivity when encountering a repetitive sequence (52). Besides, we also assessed the KF extension on the template containing a shorter (AAGGG)_4_, showing that the polymerase processivity and full-length products were almost unaffected by K^+^ concentration (Supplementary Figure S17). This observation is in line with our forementioned results that four DNA AAGGG repeats did not form a stable intramolecular G4 in 150 mM K^+^ (Figure 4A and Supplementary Figure S13). These results suggest that when the number of AAGGG repeats increases to a certain threshold, the sequence can naturally form intramolecular G4s under a physiologically relevant ionic condition to impede DNA polymerase processivity and cause replication stalling.

Given that NMM could bind and stabilize the G4 formed by AAGGG repeats (Figure 5), we also performed the *in vitro* replicational assay under various concentrations of NMM to examine the effect of ligand-induced G4 stabilization on polymerase processivity. No appreciable full-length extension product was observed under 100 μM NMM for the template containing (AAGGG)_8_ (Figure 6C, left), suggesting that the formation of thermostable G4s in AAGGG repeats had tremendous impeding effect on polymerase processivity. The DNA replication was not affected by NMM for the reference (AAAAG)_8_ template (Figure 6C, right).

### G4 formation in pathogenic AAGGG repeats reduced gene expression in cells

**Figure 7.**
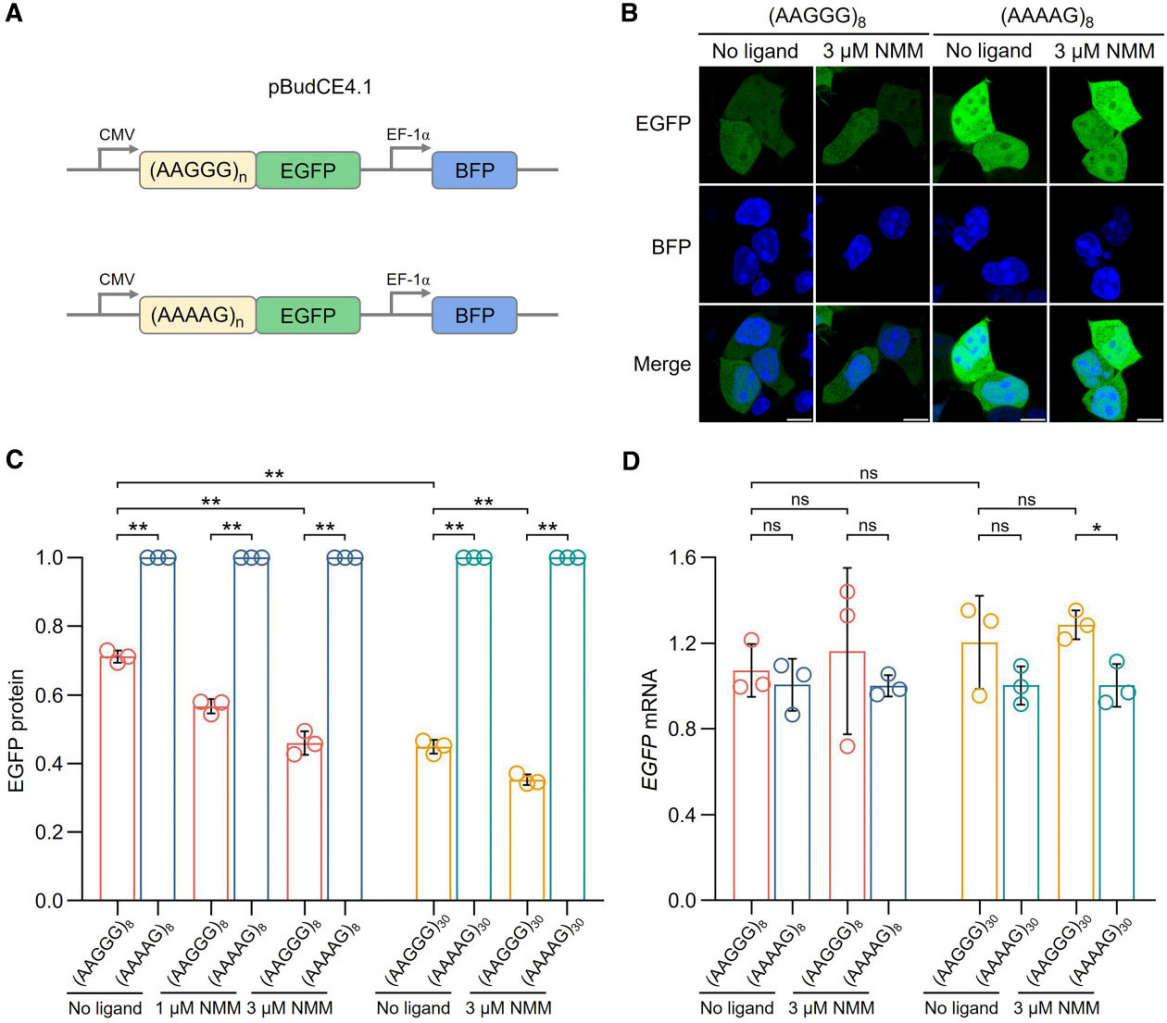
(**A**) Schematic of the live-cell gene expression reporter constructs in HEK293T cells. (**B**) Confocal microscopy (scale bars, 10 μm) of HEK293T cells expressing reporter constructs containing (AAGGG)_8_ and (AAAAG)_8_ without or with NMM treatment. (**C**, **D**) The EGFP protein and mRNA levels in HEK293T cells expressing reporter constructs containing (AAGGG)_8_ (red), (AAAAG)_8_ (blue), (AAGGG)_30_ (yellow) and (AAAAG)_30_ (green) without or with NMM treatment. Data are given as means ± SD of three independent experiments. *P < 0.05, **P < 0.01, ns: non-significant. [NMM] = 0/1/3 μM.

The protein loss-of-function with reduced RFC1 levels in CANVAS patients has been recently reported, but the underlying molecular mechanism for reduced gene expression remains unclear (9,10). We next sought to explore whether the G4 formation in AAGGG repeats can affect gene expression *in vivo*. For this aim, we engineered several live-cell gene expression reporters using a pBudCE4.1 vector-based plasmid construct that expressed EGFP under the CMV promoter serving as the gene expression reporter, and expressed BFP under the EF-1α promoter serving as an internal reference. The pathogenic AAGGG repeats or nonpathogenic AAAAG repeats with various repeat lengths (n = 8, 30) were inserted to the upstream of the EGFP coding sequence (Figure 7A).

Under confocal microscopy, the EGFP fluorescence in HEK293T cells transfected with (AAGGG)_8_ was obviously weaker than the reference (AAAAG)_8_ (Figure 7B). We further quantified the EGFP protein levels using flow cytometry based on the relative fluorescence intensity of EGFP/BFP (Figure 7C and Supplementary Figure S18). There was a significant reduction of EGFP protein level in cells transfected with (AAGGG)_8_ comparing to the reference (AAAAG)_8_ without NMM treatment (Figure 7C). To further verify that the reduced EGFP protein level was caused by G4 formation in AAGGG repeats, we treated cells with NMM which was demonstrated to stabilize G4 formation in AAGGG repeats (Figures 5C, 6C). Comparing to cells expressing the (AAGGG)_8_ construct without ligand treatment, cells expressing the (AAGGG)_8_ construct with 1 and 3 μM NMM treatment showed significantly reduced EGFP protein levels in a ligand-concentration-dependent manner (Figure 7C). Notably, the EGFP protein level in cells transfected with a longer (AAGGG)_30_ was significantly lower comparing to (AAGGG)_8_ without NMM treatment (Figure 7C). These results suggest that G4 formation in AAGGG repeats can reduce gene expression with longer repeats having a more pronounced inhibitory effect.

With a more prudent consideration, the reduced EGFP protein levels observed in the (AAGGG)_8_ and (AAGGG)_30_ groups might be caused by impaired transcription and/or translation of the reporter gene. Therefore, we also examined the *EGFP* mRNA level by RT-qPCR. The level of *EGFP* mRNA showed no significant difference between (AAGGG)_8_ and the reference (AAAAG)_8_ without NMM treatment (Figure 7D). Cells treated with 3 μM NMM also showed no significant difference in *EGFP* mRNA levels between the (AAGGG)_8_ and (AAAAG)_8_ groups. Similar phenomena were generally observed for cells transfected with longer (AAGGG)_30_ and (AAAAG)_30_ (Figure 7D). These indicate that transcription of the reporter gene was not affected by the presence of AAGGG repeats regardless of the repeat length. Therefore, the reduced EGFP protein level in the pathogenic (AAGGG)_8_ and (AAGGG)_30_ groups shown in Figure 7C can be ascribed to G4 formation that impaired the translation process.

## Discussion

Since the discovery of pathogenic *RFC1* AAGGG repeat expansion associated with CANVAS (4–7) and several other neurodegenerative diseases including the PD (11,12), MSA (13–15) and CIAP (16,17), few progress has been achieved towards a clearer understanding of molecular mechanism by which expanded AAGGG repeats exert detrimental function. Remarkably, the functional consequence of nucleic acids is not only determined by their primary sequence, but also the structural property. Here, we report that both DNA and RNA AAGGG repeats from the pathogenic *RFC1* can form parallel G4 structures, whose oligomeric states depend on repeat length and flanking residues. For the d(AAGGG)_2_ that forms a mixture of bimolecular and tetramolecular G4s in 150 mM K^+^ (Figure 1B), the tetramolecular G4 is prevented by the presence of two 3′-flanking adenine residues (Figure 1D). Our determined high-resolution NMR structure of the parallel bimolecular G4 of d(AAGGG)_2_AA shows three G-tetrad layers with extensive base-base stackings (Figure 3), providing atomic-level insights into understanding how AAGGG repeats pack into a higher-order G4 structure. As the biological relevance of G4 is often restrained to intramolecular structure, we further examine the possibility of forming intramolecular G4s in both DNA and RNA repeats. When the number of repeats increases, d(AAGGG)_8_ shows a propensity to form an intramolecular G4 as suggested by the results of 1D ^1^H NMR, CD and PAGE (Figure 4A and Supplementary Figure S14). The RNA molecule of r(AAGGG)_4_AA is found to form an intramolecular G4 as suggested by twelve well-resolved guanine H1 signals in K^+^ (Figure 4A).

Katahira *et al.* have reported noncanonical DNA G4 structures formed by the purine-rich GGA repeats. One molecule of d(GGA)_4_ folded into an intramolecular G4 composed of a G·G·G·G tetrad and a G·A·G·A·G·A·G heptad with the four G–G segments aligned parallelly, and two G4s formed a dimer stabilized through stacking interactions between the heptads (53). They further determined an intramolecular G4 structure formed by d(GGA)_8_, in which two intramolecular parallel G4s were packed in a tail-to-tail manner (54). Comparing the G4 structures of GGA and AAGGG repeats, the non-terminal adenine residues in GGA repeats constituted the heptad whereas those in AAGGG repeats located in the loop. In addition, it appeared that the purine-rich GGA and AAGGG repeats tended to form intermolecular G4s when the repeats were short (e.g. n = 4) and intramolecular G4s when the repeats became long (e.g. n = 8). The structures of nonpathogenic AAAAG repeats were not investigated in this study, as Abdi *et al.* have recently demonstrated that AAAAG repeats did not form stable secondary structures (32). The distinctive capability of pathogenic AAGGG and nonpathogenic AAAAG repeats of forming noncanonical DNA/RNA structures points to a role of G4 structure in exerting detrimental function that contributes to CANVAS pathogenesis.

**Figure 8.**
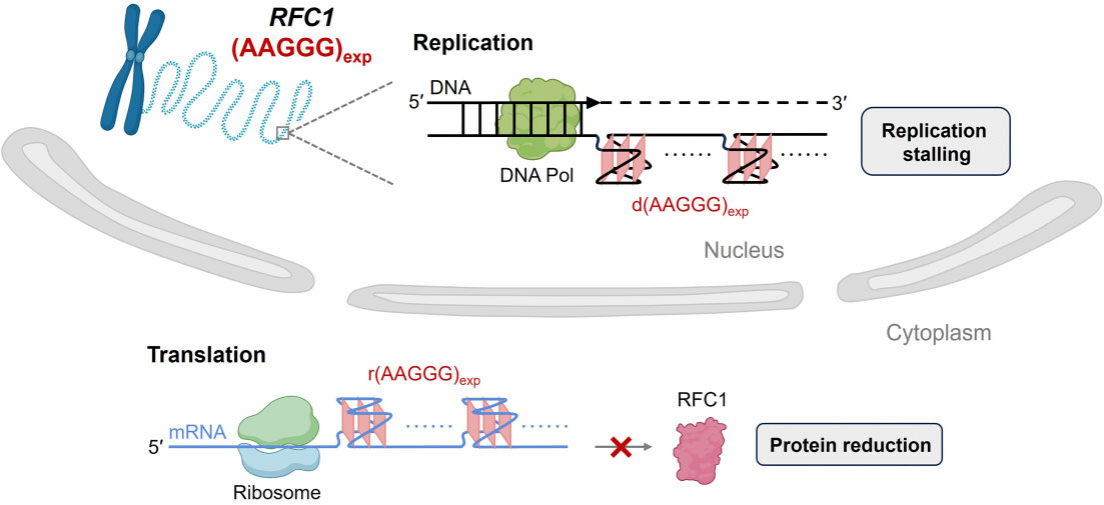
A proposed model for the functional consequence of G4 formation in the pathogenic *RFC1* AAGGG repeats. The expanded AAGGG repeats form DNA G4 structure(s) that impede polymerase processivity and cause replication stalling, and form RNA G4 structure(s) that impair translation and reduce protein production.

Recent data suggests a RFC1 protein loss-of-function mechanism caused by the homozygous *RFC1* AAGGG repeat expansion (7), but the underlying reason is puzzlingly unclear. Here, we first demonstrate that AAGGG repeats form G4 structures by high-resolution NMR spectroscopy, and then elucidate the roles of G4 structures in causing aberrant biological processes including DNA replication stalling and gene expression inhibition (Figure 8). Comparing to cells transfected with nonpathogenic (AAAAG)_8_ or (AAAAG)_30_, the protein level of reporter gene was significantly reduced in cells transfected with (AAGGG)_8_ or (AAGGG)_30_, and further decreased upon NMM treatment that promoted G4 formation (Figure 7B, C). Interestingly, the mRNA levels of reporter gene were not significantly different between the pathogenic (AAGGG)_8/30_ and nonpathogenic (AAAAG)_8/30_ groups (Figure 7D). Therefore, the reduced gene expression could be attributed to G4 formation in AAGGG repeats that impaired the translation but not transcription process. It has been well documented that the G4 structures formed in mRNAs can downregulate gene expression by preventing the binding of desired proteins or recruiting the binding of undesired proteins, which result in errored RNA splicing and impaired translation (55–58). Besides, RNA transcripts containing expanded repeats can form unusual structures to sequester RNA-binding proteins and form nuclear foci in repeat expansion diseases (44,59,60). Our results thus provide an important structural basis for understanding the roles of G4 in dysregulating gene expression and cellular dysfunctions in CANVAS and the other relevant neurodegenerative diseases.

All repeat expansion diseases identified so far manifest only beyond a critical number of repeats (1), although a correlation between the AAGGG repeat length and clinical onset or progression of CANVAS has yet to be established. The present work focuses on the structures of short AAGGG repeats from the pathogenic *RFC1* gene associated with CANVAS, and we have not examined the structures of disease relevant repeats (400–2000 repeats) due to technical limitations. Nonetheless, it has been well perceived that the high-resolution structures of short repeats can provide valuable information to assist the structural study of longer repeats using more appropriate methods such as chemical probing (61–63) and single-molecule technique (64,65). In addition, we found that AAGGG repeats inhibited gene expression in a repeat-length-dependent manner, i.e. cells transfected with (AAGGG)_30_ had a more pronounced effect on reducing the protein level of reporter gene than (AAGGG)_8_ (Figure 7). It can be anticipated that the pathogenic *RFC1* harboring hundreds to thousands of AAGGG repeats may exert a more detrimental function to disease pathogenesis. Recently, the importance of G4-resolvases and G4-unfolding ligands has been increasingly recognized for neurodegenerative disease therapy (66–69). For instance, the Cockayne Syndrome B protein and DEAH-Box helicase 9 could resolve DNA G4 structures to restore normal cellular activities (66,67). Therefore, the high-resolution structural information provided by this work will facilitate the discovery or rational design of helicases and ligands that can tackle the culpable G4 structures.

## Conclusion

In sum, we have demonstrated the formation of DNA and RNA parallel G4 structures in the pathogenic *RFC1* AAGGG repeats by providing a thorough high-resolution NMR structural investigation. We also elucidated an unprecedent role of G4 in aberrant biological processes, including replication stalling and downregulated gene expression, with a more pronounced effect by longer AAGGG repeats. This work for the first time provides a structural basis for understanding the molecular mechanism by which *RFC1* AAGGG repeat expansion elicits detrimental function to the development of disease. Furthermore, the high-resolution G4 structure resolved in this study will facilitate rational design of helicases and drugs targeting the G4 of AAGGG repeats for therapeutic interventions.

## Supplementary Material

gkae032_Supplemental_File

## Data Availability

The coordinates and NMR chemical shifts of the bimolecular G4 structure of d(AAGGG)_2_AA have been deposited to the Protein Data Bank (PDB ID: 8X1V) and Biological Magnetic Resonance Bank (BMRB ID: 36612), respectively.
