# Supplementary material for: Structural investigation of pathogenic RFC1 AAGGG pentanucleotide repeats reveals a role of G-quadruplex in dysregulated gene expression in CANVAS
Source: Nucleic Acids Res. 2024 Jan 24;52(5):2698–710. doi: 10.1093/nar/gkae032 (PMC10954463; doi:10.1093/nar/gkae032)
Supplement: gkae032_Supplemental_File [file gkae032_supplemental_file.pdf]

Supporting Information

Structural Investigation of Pathogenic *RFC1* AAGGG Pentanucleotide Repeats Reveals a Role of G-Quadruplex in Dysregulated Gene Expression in CANVAS

Yang Wang[1,2]†, Junyan Wang[2]†, Zhenzhen Yan[3], Jianing Hou[4], Liqi Wan[2], Yingquan Yang[3], Yu Liu[2], Jie Yi[2], Pei Guo[2]*, Da Han[2,4]*

[1] School of Materials Science and Engineering, Tianjin University, Tianjin 300350, China

[2] Zhejiang Cancer Hospital, Hangzhou Institute of Medicine (HIM), Chinese Academy of Sciences, Hangzhou, Zhejiang 310022, China

[3] School of Biology and Biological Engineering, South China University of Technology, Guangzhou, Guangdong 510006, China

[4] Institute of Molecular Medicine (IMM) Renji Hospital, School of Medicine, Shanghai Jiao Tong University, Shanghai 200127, China

* To whom correspondence should be addressed. Email: dahan@sjtu.edu.cn; guopei@ibmc.ac.cn

† The authors wish it to be known that, in their opinion, the first two authors should be regarded as Joint First Authors.

Table S1. DNA and RNA sequences used in this study.

Name	Sequence
DNA	
d(AAGGG)$_2$	5′-AAGGGAAGGG-3′
d(AAGGG)$_2$A	5′-AAGGGAAGGGA-3′
d(AAGGG)$_2$AA	5′-AAGGGAAGGGAA-3′
d(AAGGG)$_2$AA-G3iso	5′-AA$\underline{^{15N}G}$GGAAGGGAA-3′
d(AAGGG)$_2$AA-G4iso	5′-AAG$\underline{^{15N}G}$GAAGGGAA-3′
d(AAGGG)$_2$AA-G5iso	5′-AAGG$\underline{^{15N}G}$AAGGGAA-3′
d(AAGGG)$_2$AA-G8iso	5′-AAGGGAA$\underline{^{15N}G}$GGAA-3′
d(AAGGG)$_2$AA-G9iso	5′-AAGGGAAG$\underline{^{15N}G}$GAA-3′
d(AAGGG)$_2$AA-G10iso	5′-AAGGGAAGG$\underline{^{15N}G}$AA-3′
d(AAGGG)$_4$AA	5′-AAGGGAAGGGAAGGGAAGGGAA-3′
d(AAGGG)$_8$	5′-AAGGGAAGGGAAGGGAAGGGAAGGGAAGGGAAGGGAAGGG-3′
TAG	5′-TAGGGTTAGGGT-3′
T30177	5′-GTGGTGGGTGGGTGGGT-3′

10-bp duplex ladder	5′-CGCGATCGCG-3′
20-bp duplex ladder	5′-CGCTAGCGAATTCGCTAGCG-3′
Replication primer	5′-cy5-TAATACGACTCACTATA-3′
Replication template-AAAAG8	5′-AAAAGAAAAGAAAAGAAAAGAAAAGAAAAGAAAAGAAAAGTATAGTGAGTCGTATTA-3′
Replication template-AAGGG8	5′-AAGGGAAGGGAAGGGAAGGGAAGGGAAGGGAAGGGAAGGGTATAGTGAGTCGTATTA-3′
qPCR primer BFP-F	5′-AAGCTGTACATGGAGGGCAC-3′
qPCR primer BFP-R	5′-TGAAGGTCTTGCTGCCGTAG-3′
qPCR primer EGFP-F	5′-GACGTAAACGGCCACAAGTT-3′
qPCR primer EGFP-R	5′-AAGTCGTGCTGCTTCATGTG-3′
RNA	
r(AAGGG)$_4$AA	5′-AAGGGAAGGGAAGGGAAGGGAA-3′

Table S2. ^{1}H chemical shifts of d(AAGGG)$_2$AA (ppm).

Residue	H1	H2	H8	H1′	H2′	H2″	H3′	H4′	H5′	H5″
A1	-	7.674	7.702	5.971	2.254	2.404	4.686	3.985	3.445	3.445
A2	-	7.721	7.960	5.778	2.692	2.692	4.925	[a]	[a]	[a]
G3	11.679	-	8.002	6.104	2.767	2.986	5.038	4.511	4.172	4.172
G4	11.342	-	7.570	6.045	2.547	2.676	4.904	4.460	4.254	4.309
G5	11.176	-	7.682	6.326	2.608	2.542	4.979	[a]	[a]	[a]
A6	-	8.307	8.466	6.386	2.781	2.781	4.965	4.261	[a]	[a]
A7	-	8.246	8.504	6.601	2.941	2.941	5.189	4.622	4.329	4.329
G8	11.324	-	7.853	6.010	2.397	2.868	4.912	4.447	3.801	4.136
G9	11.299	-	7.896	5.970	2.741	2.741	5.056	4.519	4.238	4.238
G10	10.801	-	7.590	5.884	2.408	2.629	4.957	4.454	[a]	[a]
A11	-	7.308	7.847	5.916	2.296	2.442	4.852	4.280	4.118	4.118
A12	-	7.488	8.021	5.996	2.447	2.299	4.556	4.081	4.006	4.006

[a] Resonances could not be unambiguously assigned due to weak $^3J_{H3'-H4'}$ and $^3J_{H4'-H5'/H5''}$ couplings or signal overlaps.
The chemical shifts of labile protons (G H1) and non-labile protons (H2/H8/H1′/H2′/H2″/H3′/H4′/H5′/H5″) were measured from 2D NOESY spectra acquired at 15 °C and 25 °C, respectively.

Table S3. NOE-derived distance restraints (intranucleotide/internucleotide: 140/108) for structural calculation of d(AAGGG)$_2$AA.

Residue	A1	A2	G3	G4	G5	A6	A7	G8	G9	G10	A11	A12	A13	A14	G15	G16	G17	A18	A19	G20	G21	G22	A23	A24
A1	5	7	-	-	-	-	-	-	-	-	-	-	-	-	-	-	-	-	-	-	-	-	-	-
A2		4	5	-	-	-	-	-	-	-	-	-	-	-	-	-	-	-	-	-	-	-	-	-
G3			10	8	-	-	-	-	-	-	-	-	-	-	-	-	-	-	-	-	-	-	-	-
G4				9	5	-	-	-	-	-	-	-	-	-	-	-	-	-	-	-	-	-	-	-
G5					7	1	1	-	-	-	-	-	-	-	-	-	-	-	-	-	-	-	-	-
A6						5	10	4	-	-	-	-	-	-	-	-	-	-	-	-	-	-	-	-
A7							5	-	-	-	-	-	-	-	-	-	-	-	-	-	-	-	-	-
G8								5	3	-	-	-	-	-	-	-	-	-	-	-	-	-	-	-
G9									1	6	-	-	-	-	-	-	-	-	-	-	-	-	-	-
G10										7	3	-	-	-	-	-	-	-	-	-	-	-	-	-
A11											5	1	-	-	-	-	-	-	-	-	-	-	-	-
A12												7	-	-	-	-	-	-	-	-	-	-	-	-
A13													5	7	-	-	-	-	-	-	-	-	-	-
A14														4	5	-	-	-	-	-	-	-	-	-
G15															10	8	-	-	-	-	-	-	-	-
G16																9	5	-	-	-	-	-	-	-
G17																	7	1	1	-	-	-	-	-
A18																		5	10	4	-	-	-	-
A19																			5	-	-	-	-	-
G20																				5	3	-	-	-
G21																					1	6	-	-
G22																						7	3	-
A23																							5	1
A24																								7

Table S4. Sugar dihedral angles (H1′-C1′-C2′-H2′) and glycosidic torsion angle (χ) restraints for structural calculation of d(AAGGG)$_2$AA.

Residue	H1′-C1′-C2′-H2′ (°)	χ (°)
A1	-	90-330
A2	-	90-330
G3	145-175	90-330
G4	140-170	90-330
G5	-	90-330
A6	-	90-330
A7	-	90-330
G8	-	90-330
G9	-	90-330
G10	-	90-330
A11	-	90-330
A12	-	90-330
A13	-	90-330
A14	-	90-330
G15	145-175	90-330
G16	140-170	90-330
G17	-	90-330
A18	-	90-330
A19	-	90-330
G20	-	90-330
G21	-	90-330
G22	-	90-330
A23	-	90-330
A24	-	90-330

Table S5. G-tetrad planarity restraints for structural calculation of d(AAGGG)$_2$AA.[a]

Atoms	Angle (°)
N9, N7, N1, N3	355-365
C5, C2, N7, C4	355-365
C2, C8, C2, C8	355-365

[a] Three planarity restraints were applied for every two adjacent guanines in each G-tetrad.

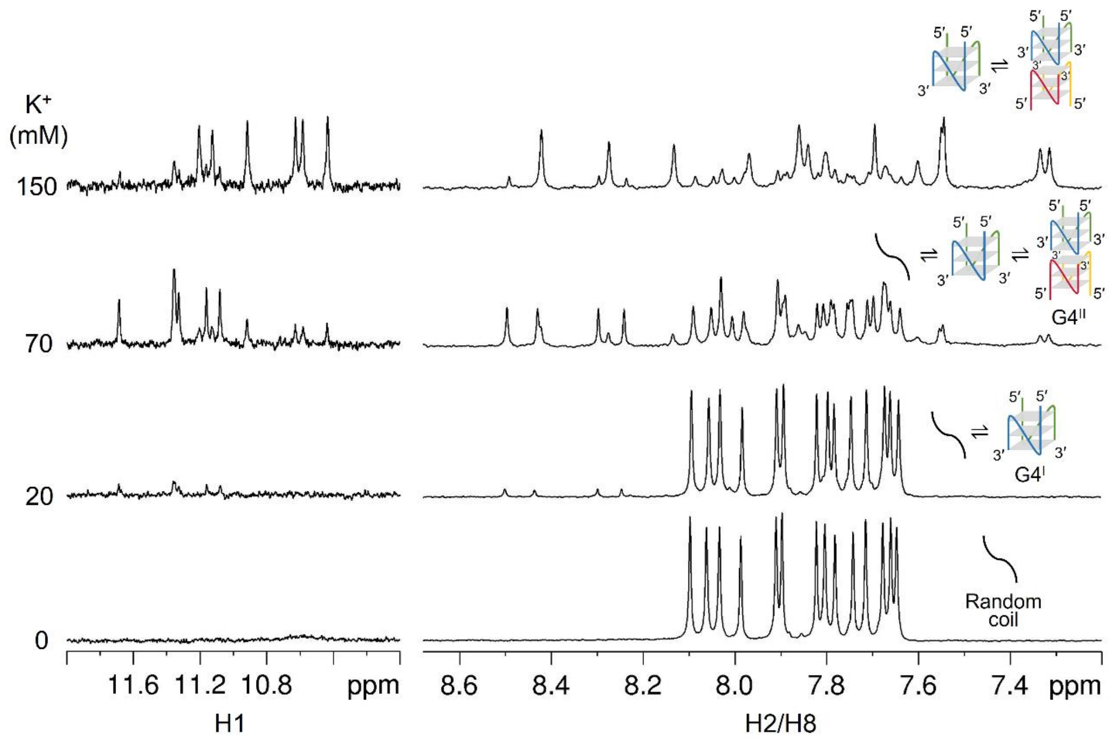

Figure S1. The 1D ^{1}H NMR spectra (imino and aromatic proton regions) of d(AAGGG)$_2$. [DNA] = 100 μM, [NaPi, pH 7] = 1 mM, [KCl] = 0/20/70/150 mM, 10% D_2O, 25 °C.

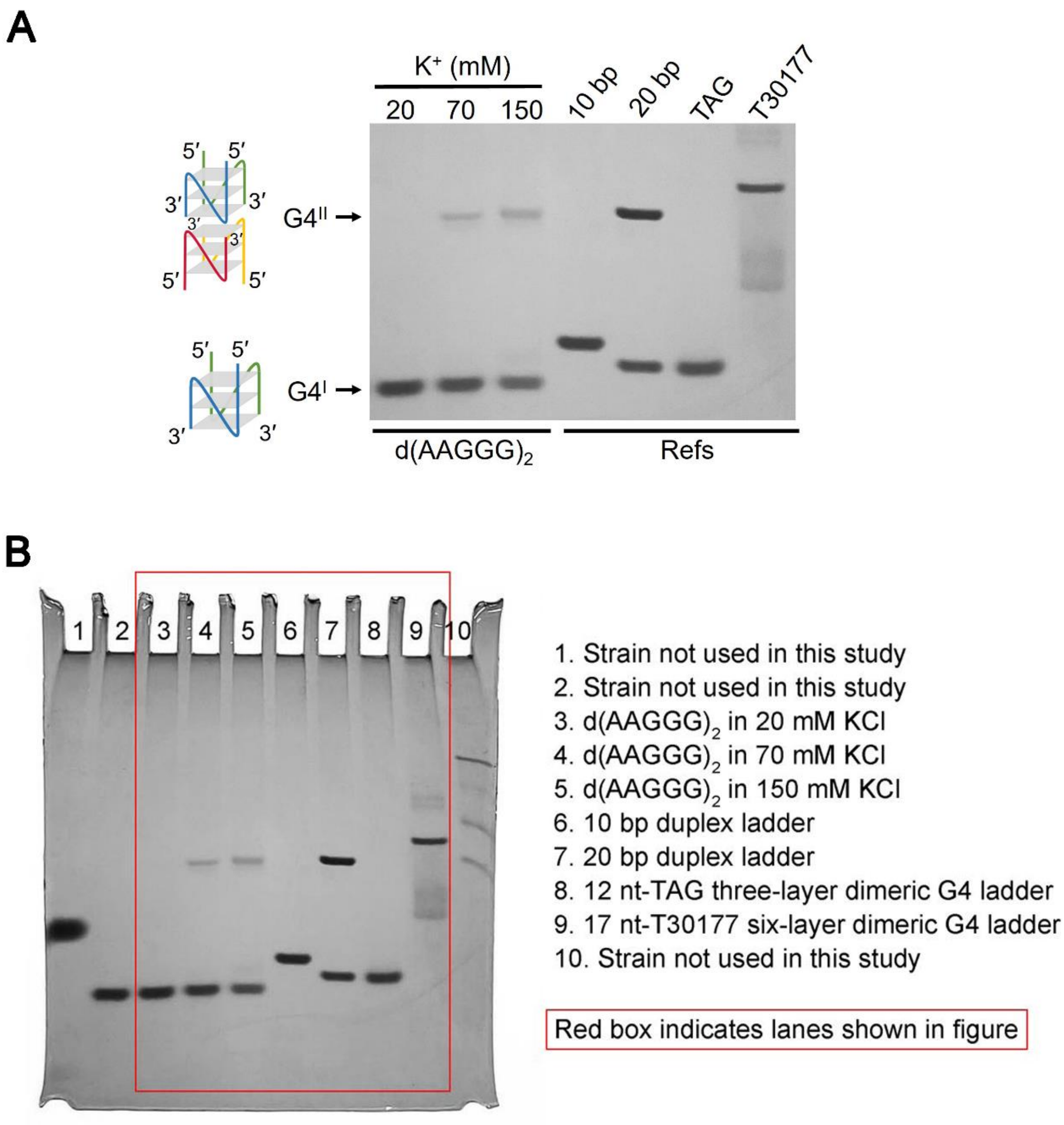

Figure S2. (A) Native PAGE of d(AAGGG)$_2$. [DNA] = 100 μM, [NaPi, pH 7] = 1 mM, 25 °C. [KCl] = 20/70/150 mM for the d(AAGGG)$_2$, [$MgCl_2$] = 10 mM for the 10-bp and 20-bp duplex references, [KCl] = 90 mM for the reference of TAG that formed a three-layer dimeric G4 (12 nt × 2), and [KCl] = 70 mM for the reference of T30177 that formed a six-layer dimeric G4 (17 nt × 2) (1, 2). The 20-bp ladder formed a 20-bp duplex structure (upper band) and a 20-nt monomeric hairpin structure (lower band) during electrophoresis. (B) Uncropped original gel for (A).

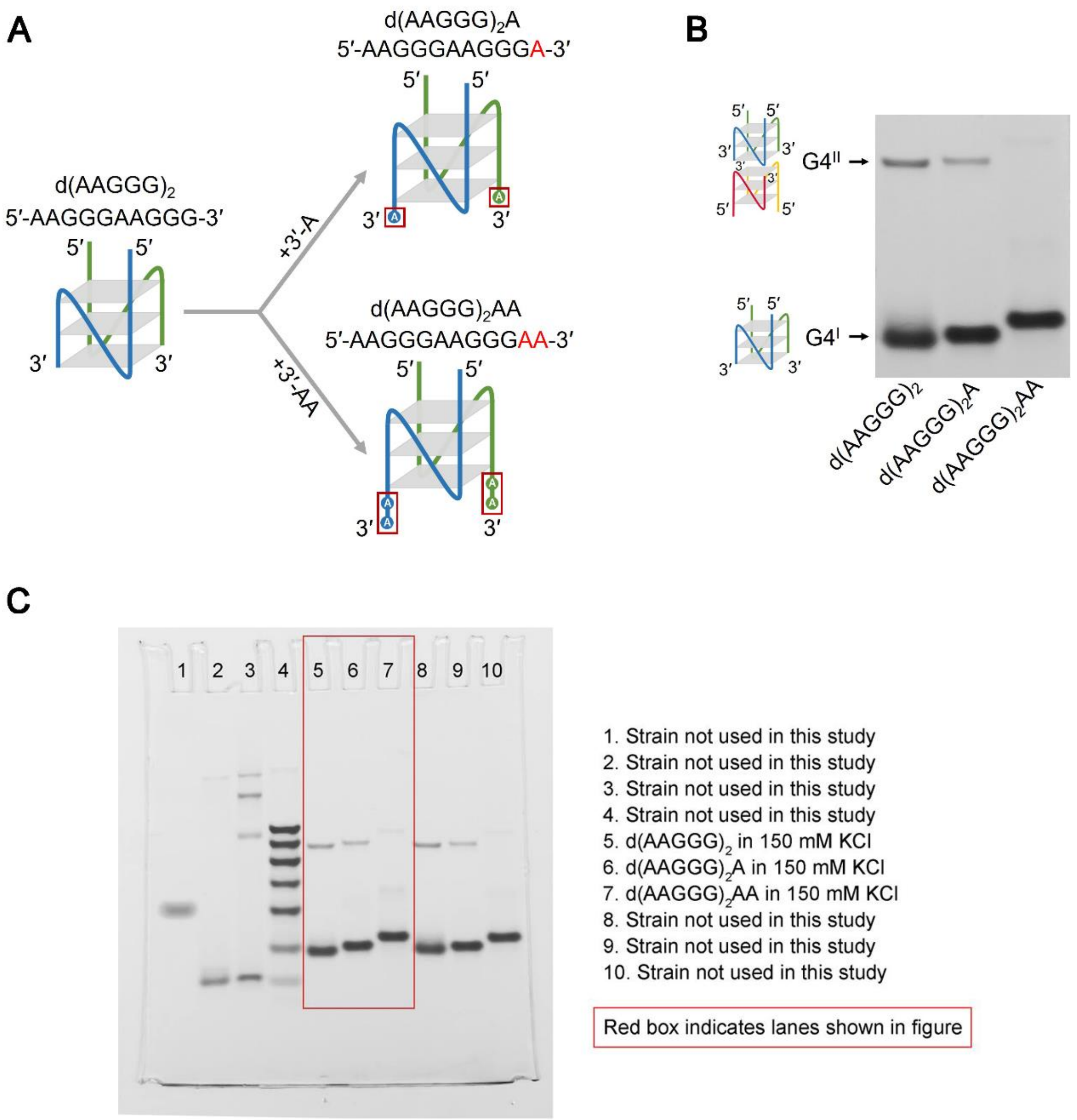

Figure S3. Effect of 3′-adenine residue(s) to d(AAGGG)$_2$ to prevent the formation of tetrameric G4. (A) Schematic of adding 3′-adenine residue(s) to d(AAGGG)$_2$. (B) Native PAGE of d(AAGGG)$_2$, d(AAGGG)$_2$A and d(AAGGG)$_2$AA. [DNA] = 100 µM, [NaPi, pH 7] = 1 mM, [KCl] = 150 mM, 25 °C. (C) Uncropped original gel.

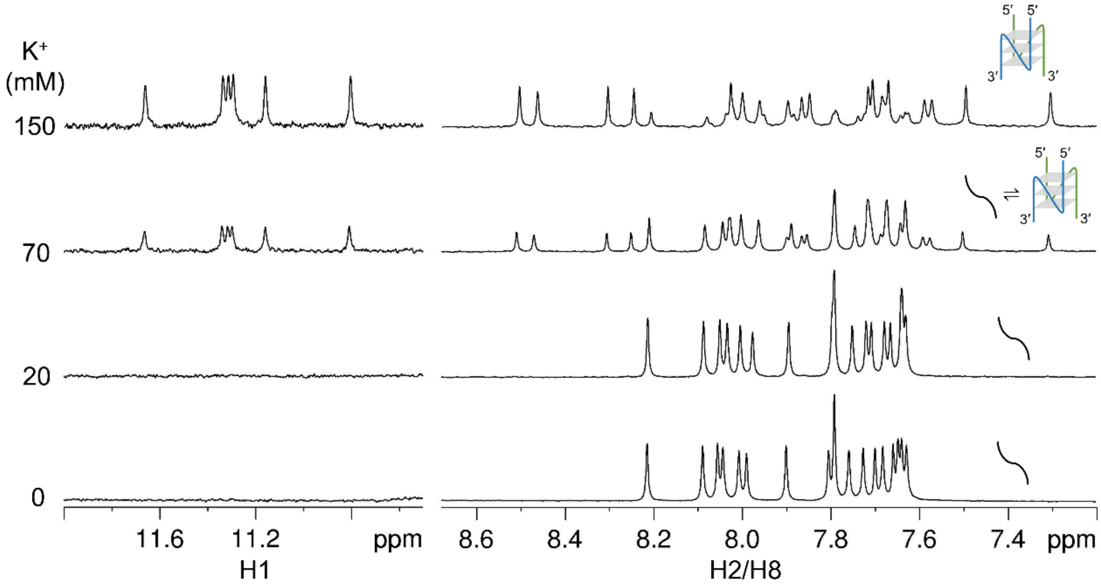

Figure S4. The 1D ^{1}H NMR spectra (imino and aromatic proton regions) of d(AAGGG)$_2$AA. [DNA] = 100 µM, [NaPi, pH 7] = 1 mM, [KCl] = 0/20/50/70/150 mM, 10% D_2O, 25 °C.

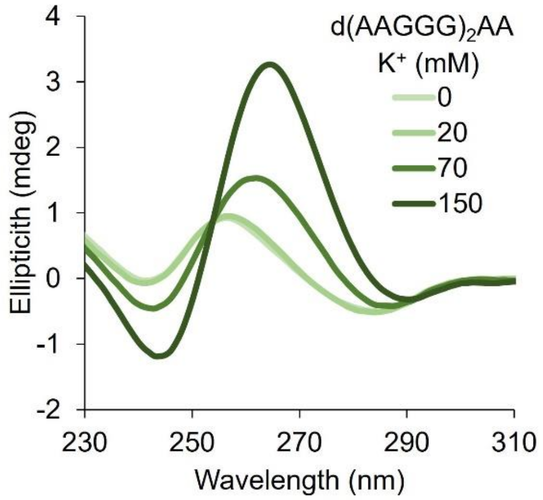

Figure S5. CD spectra of d(AAGGG)$_2$AA. [DNA] = 20 μM, [NaPi, pH 7] = 1 mM, [KCl] = 0/20/70/150 mM, 25 °C.

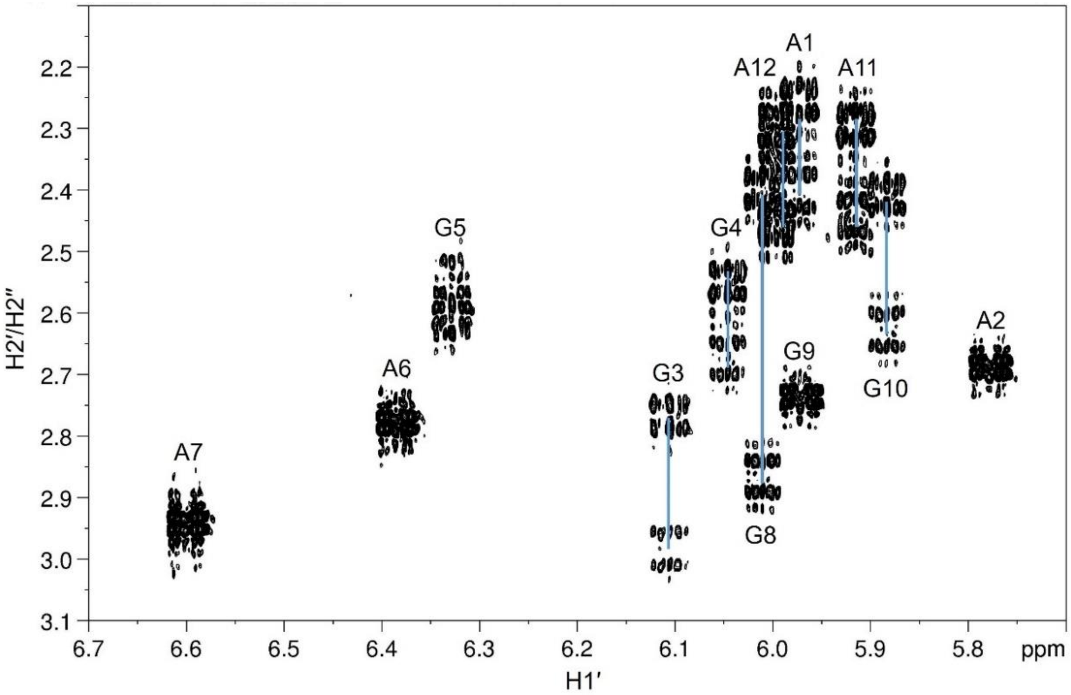

Figure S6. DQF-COSY spectrum of d(AAGGG)$_2$AA shows H1'-H2'/H2" correlation peaks. [DNA] = 800 μM, [NaPi, pH 7] = 1 mM, [KCl] = 150 mM, 99.96% D_2O, 25 °C.

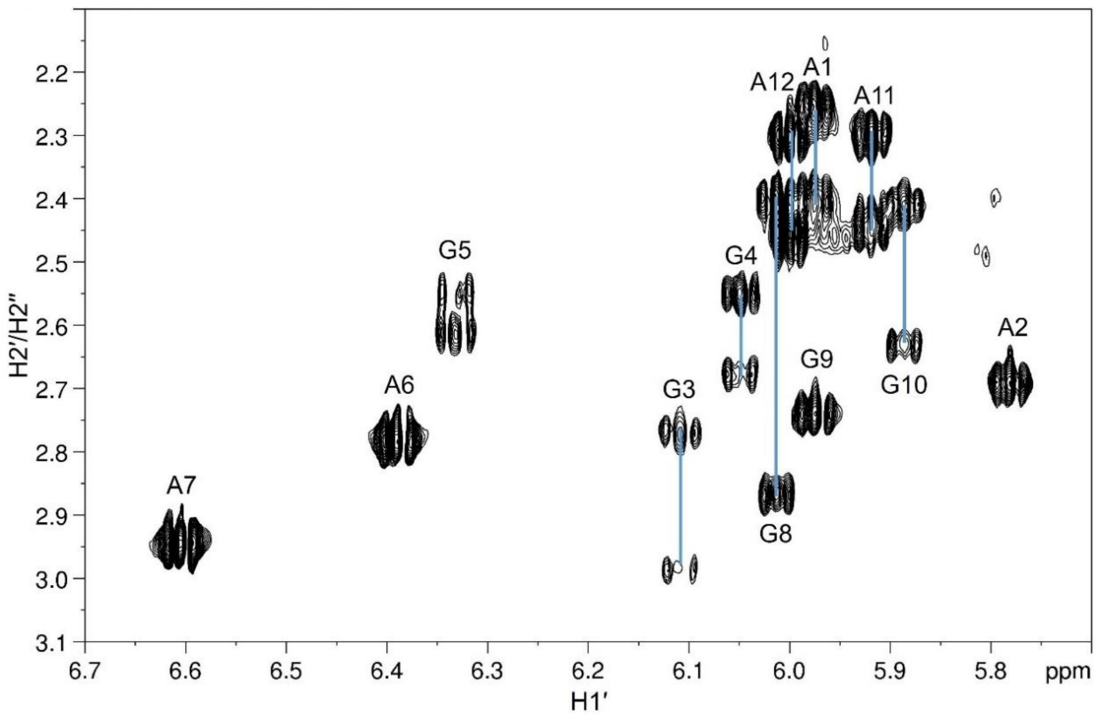

Figure S7. TOCSY spectrum of d(AAGGG)$_2$AA shows H1'-H2'/H2" correlation peaks. [DNA] = 800 μM, [NaPi, pH 7] = 1 mM, [KCl] = 150 mM, 99.96% D_2O, 25 °C.

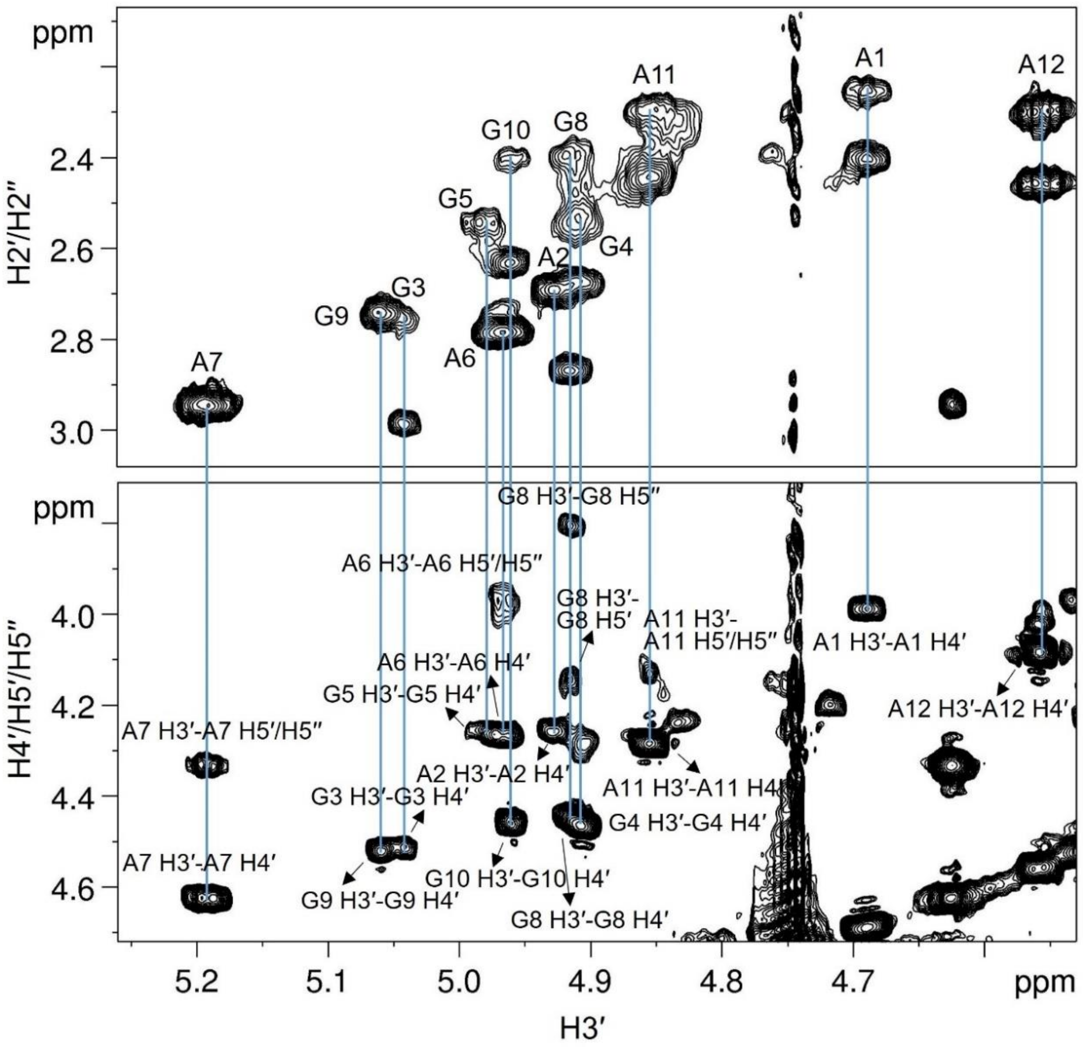

Figure S8. TOCSY spectra of d(AAGGG)$_2$AA shows H3' resonance assignment. [DNA] = 800 μM, [NaPi, pH 7] = 1 mM, [KCl] = 150 mM, 99.96% D_2O, 25 °C.

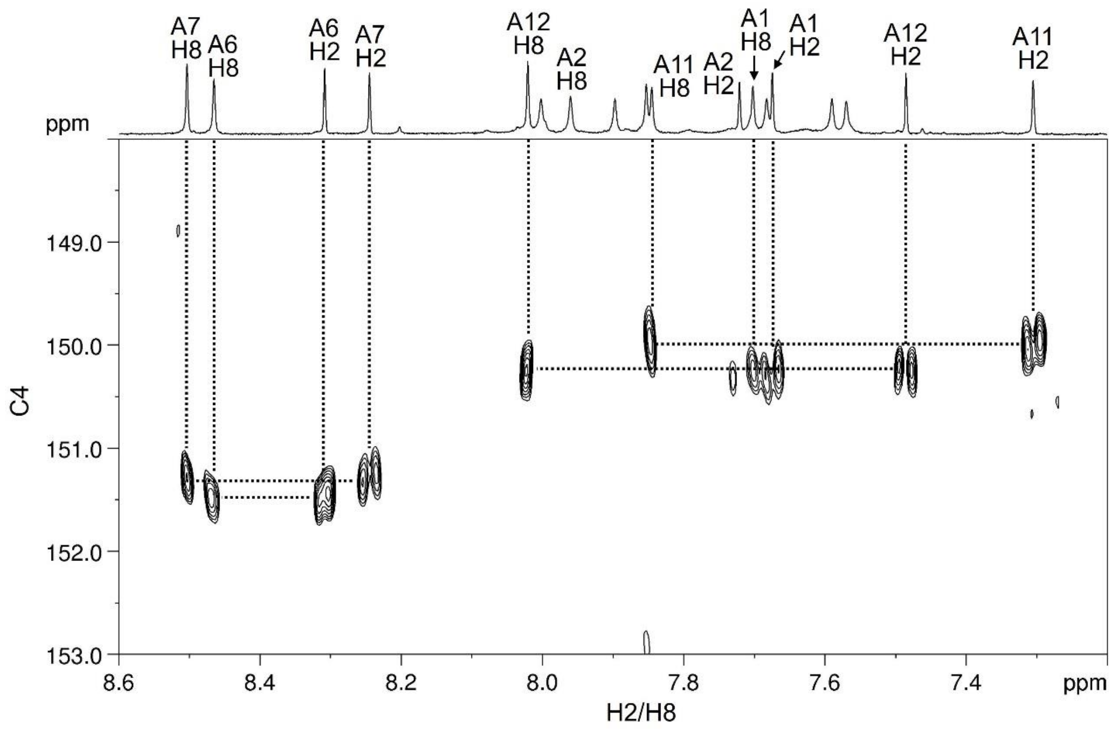

Figure S9. ^{1}H-^{13}C HMBC NMR spectrum of d(AAGGG)$_2$AA. [DNA] = 800 μM, [NaPi, pH 7] = 1 mM, [KCl] = 150 mM, 99.96% D_2O, 25 °C.

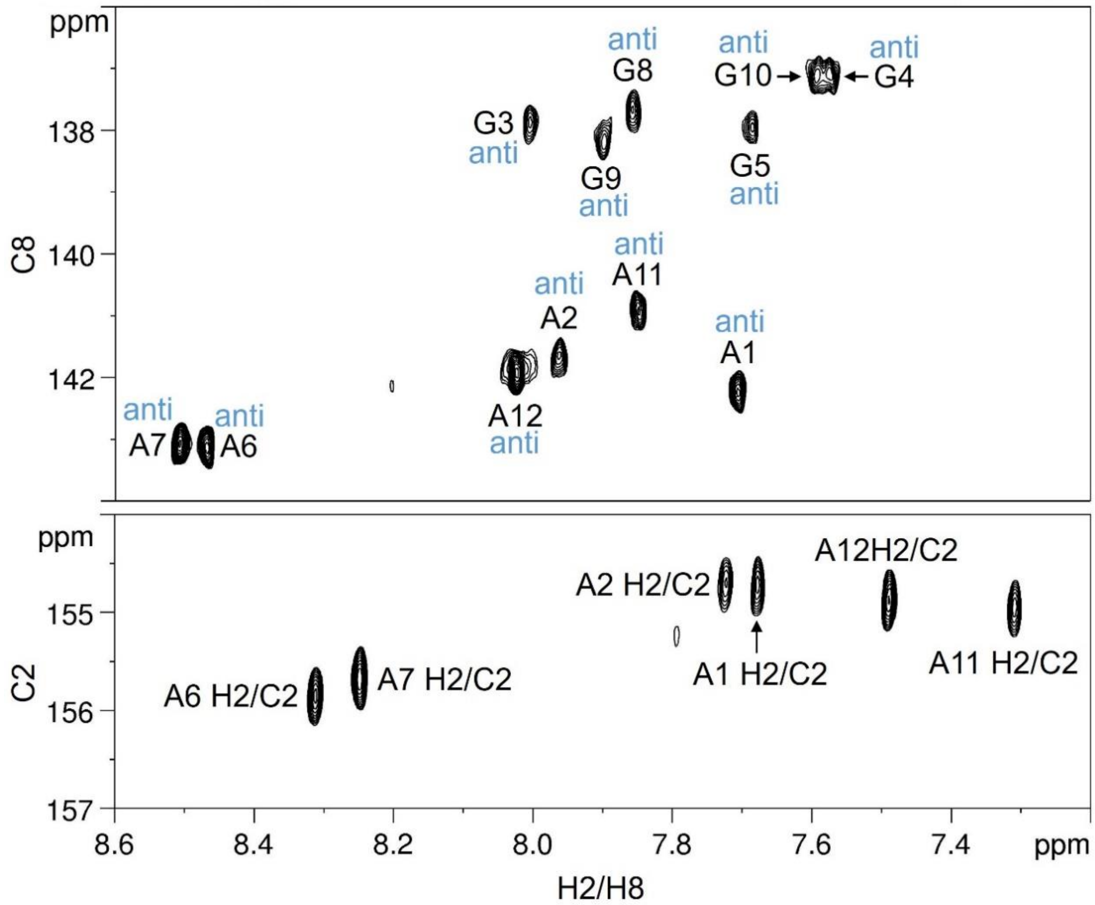

Figure S10. ^{1}H-^{13}C HSQC NMR spectrum of d(AAGGG)$_2$AA to determine *anti* or *syn* base orientation of adenine and guanine residues. [DNA] = 800 μM, [NaPi, pH 7] = 1 mM, [KCl] = 150 mM, 99.96% D_2O, 25 °C.

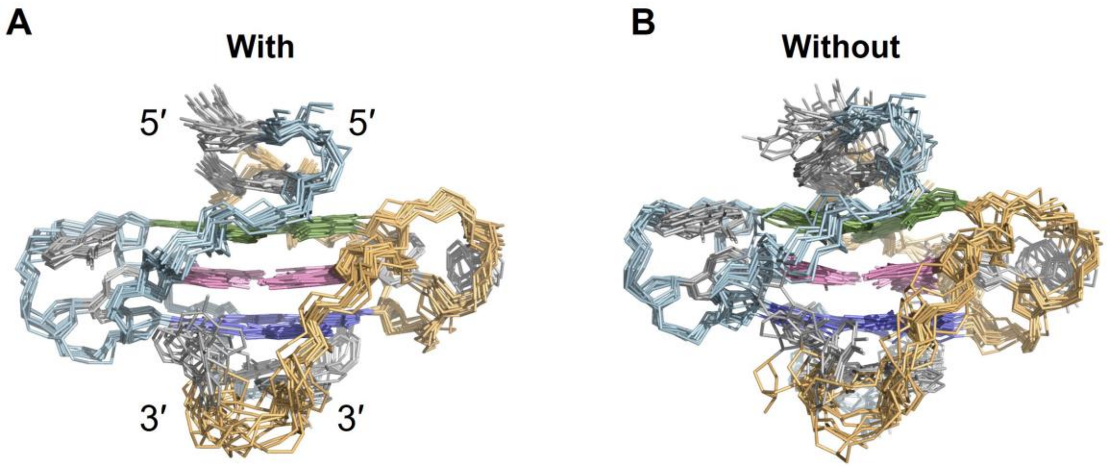

Figure S11. Superimposed 10 representative solution NMR structures of d(AAGGG)$_2$AA calculated (A) using G-tetrad planarity restraints, and (B) without using G-tetrad planarity restraints. The heavy atom RMSD values are 0.5 ± 0.1 and 1.0 ± 0.2 Å for the G-tetrad core and all residues, respectively, in (A). The heavy atom RMSD values are 0.8 ± 0.3 and 1.5 ± 0.3 Å for the G-tetrad core and all residues, respectively, in (B). The 10 structures shown in (A) are equivalent to those shown in Figure 3A of the main text. The 10 structures shown in (B) are the lowest total energy structures out of 500 independent calculations.

A

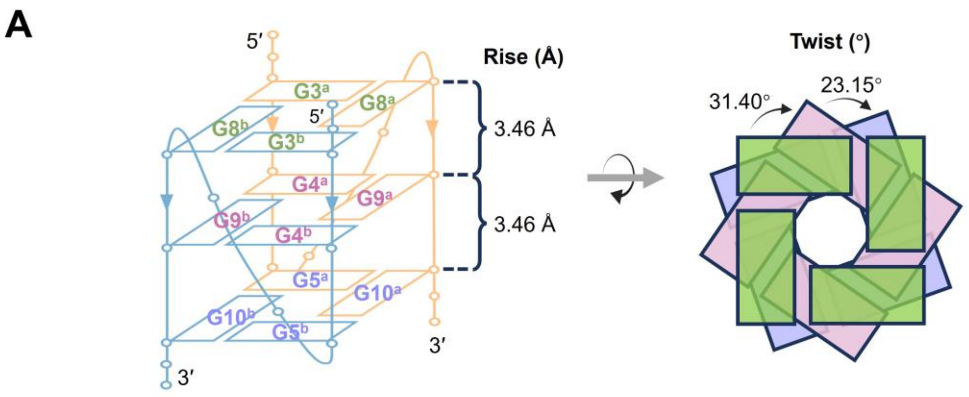

B

$G3^a{\cdot}G8^a{\cdot}G3^b{\cdot}G8^b$ | $G4^a{\cdot}G9^a{\cdot}G4^b{\cdot}G9^b$

Structures	Twist (°)	Rise (Å)
1	34.43	3.45
2	32.70	3.42
3	30.09	3.47
4	29.48	3.50
5	30.39	3.45
6	31.97	3.48
7	31.59	3.41
8	31.24	3.45
9	31.30	3.45
10	30.78	3.48
Average	31.40	3.46

$G4^a{\cdot}G9^a{\cdot}G4^b{\cdot}G9^b$ | $G5^a{\cdot}G10^a{\cdot}G5^b{\cdot}G10^b$

Structures	Twist (°)	Rise (Å)
1	21.21	3.46
2	22.67	3.52
3	23.28	3.39
4	23.90	3.45
5	24.11	3.48
6	21.88	3.47
7	25.28	3.48
8	24.04	3.46
9	23.54	3.47
10	21.57	3.39
Average	23.15	3.46

Figure S12. (A) The schematic of bimolecular parallel G4 of $d(AAGGG)_2AA$ with detailed structural information about raise and twist between every two adjacent G-tetrads. (B) The twist and rise values in each of the 10 representative solution NMR structures of $d(AAGGG)_2AA$ were analyzed using the WebTetrado tool (3).

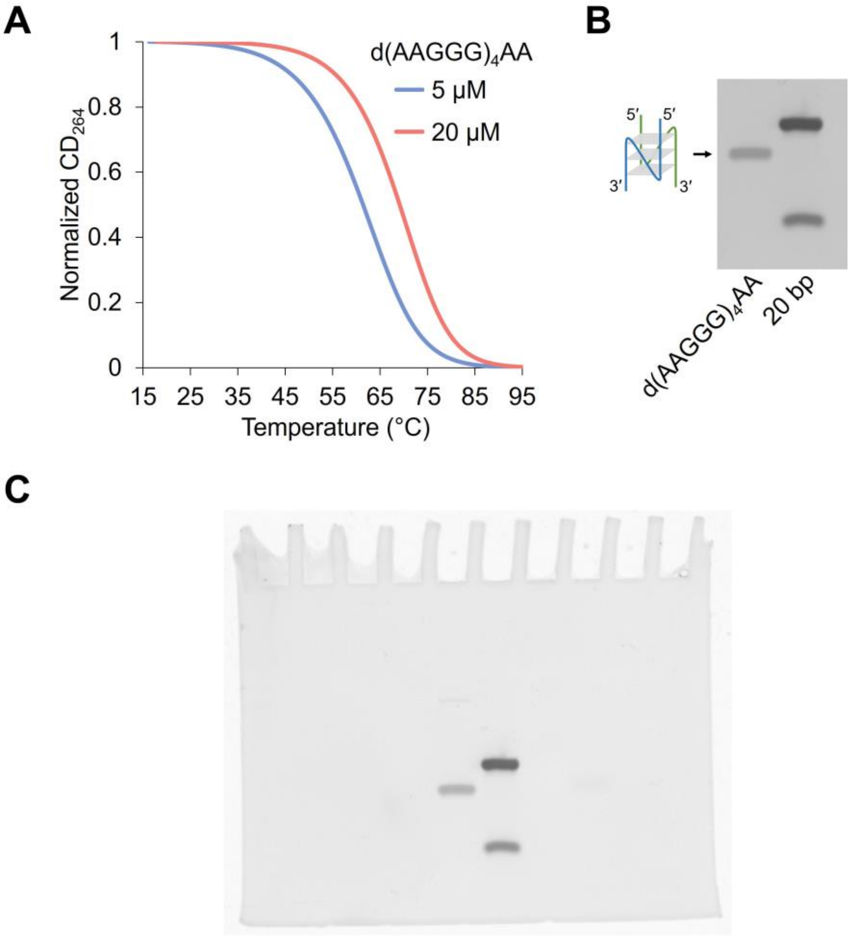

Figure S13. (A) CD melting curves of d(AAGGG)$_4$AA at 5 and 20 μM DNA concentrations. The T_m of d(AAGGG)$_4$AA was determined to be 61.4 and 69.2 °C at 5 and 20 μM DNA concentrations, respectively. (B) Native PAGE of d(AAGGG)$_4$AA. [DNA] = 100 μM, [NaPi, pH 7] = 1 mM, 25 °C. [KCl] = 150 mM for the d(AAGGG)$_4$AA, [$MgCl_2$] = 10 mM for the 20-bp duplex reference. The 20-bp ladder formed a 20-bp duplex structure (upper band) and a 20-nt monomeric hairpin structure (lower band) during electrophoresis. (C) Uncropped original gel for (B).

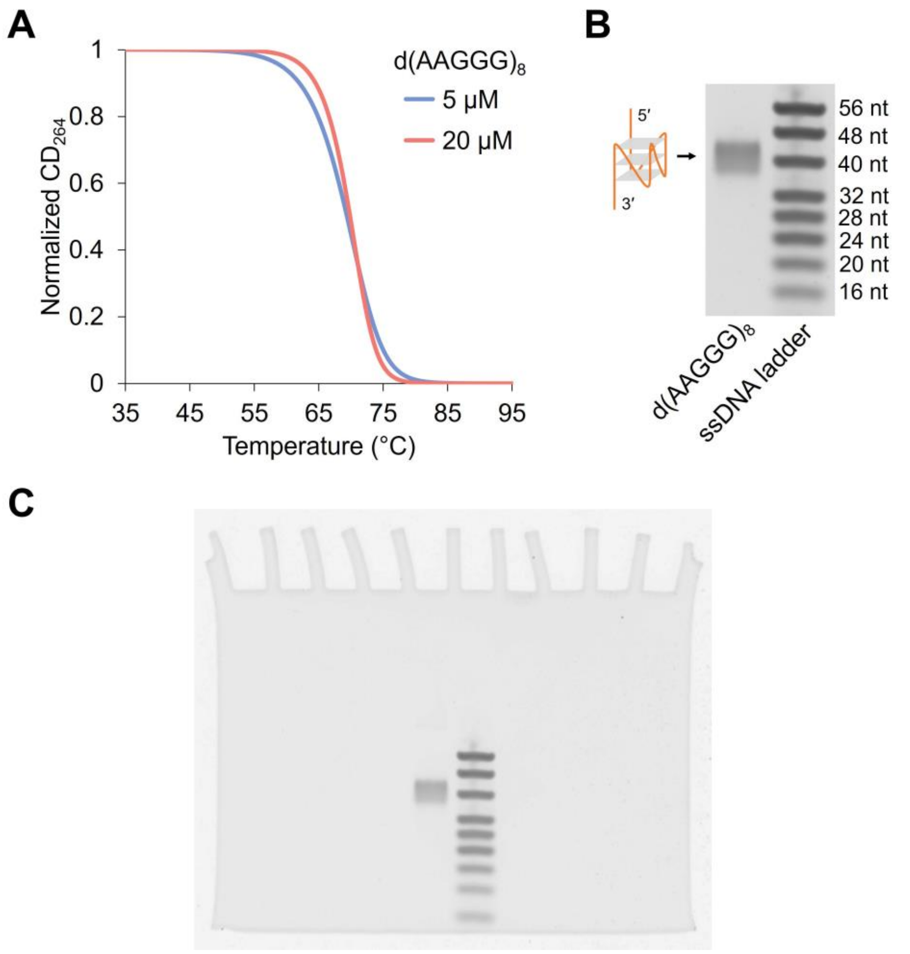

Figure S14. (A) CD melting curves of d(AAGGG)$_8$ at 5 and 20 μM DNA concentrations. The T_m of d(AAGGG)$_8$ was determined to be 69.4 and 69.8 °C at 5 and 20 μM DNA concentrations, respectively. (B) Native PAGE of d(AAGGG)$_8$. [DNA] = 100 μM, [NaPi, pH 7] = 1 mM, [KCl] = 150 mM, 25 °C. The single-stranded DNA (ssDNA) ladder was composed of nine DNA sequences corresponding to 12, 16, 20, 24, 28, 32, 40, 48 and 56-nt oligonucleotides, as stated previously (4). The ssDNA ladder was prepared to contain 0.1 mM DNA (single-strand concentration for each sequence) in 1 mM NaPi (pH 7). (C) Uncropped original gel for (B).

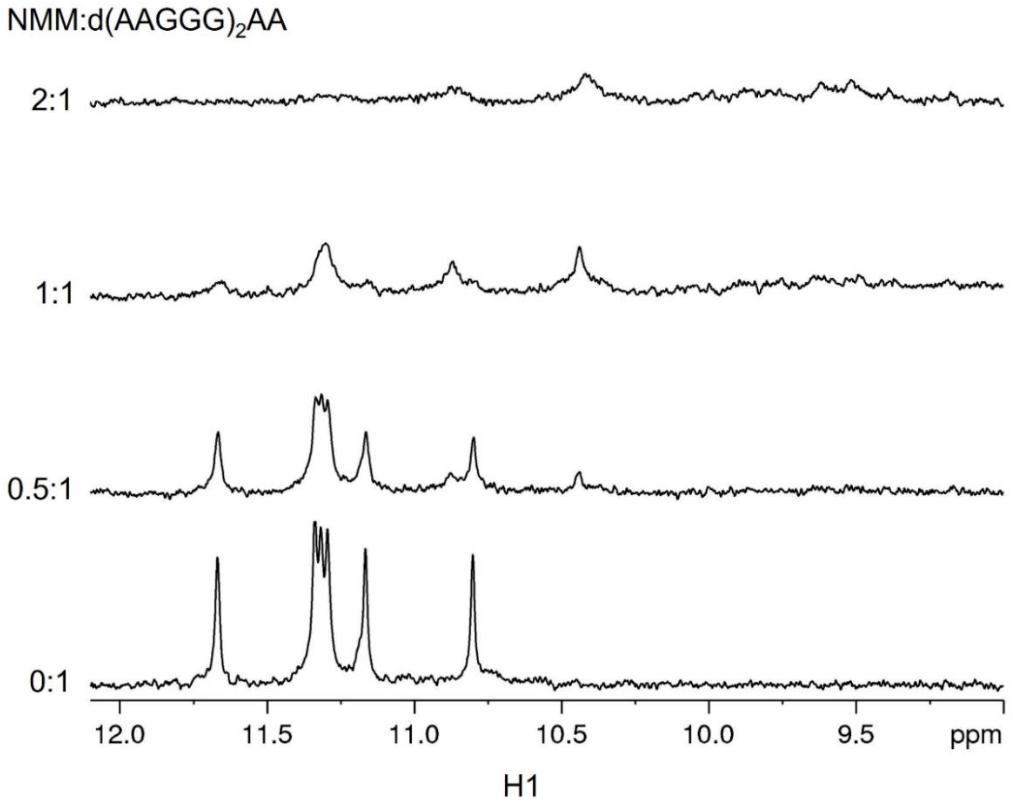

Figure S15. The 1D ^{1}H NMR spectra (imino proton regions) of NMM titration to d(AAGGG)$_2$AA. [DNA] = 100 μM, [NaPi, pH 7] = 1 mM, [KCl] = 150 mM, 10% D_2O, 25 °C.

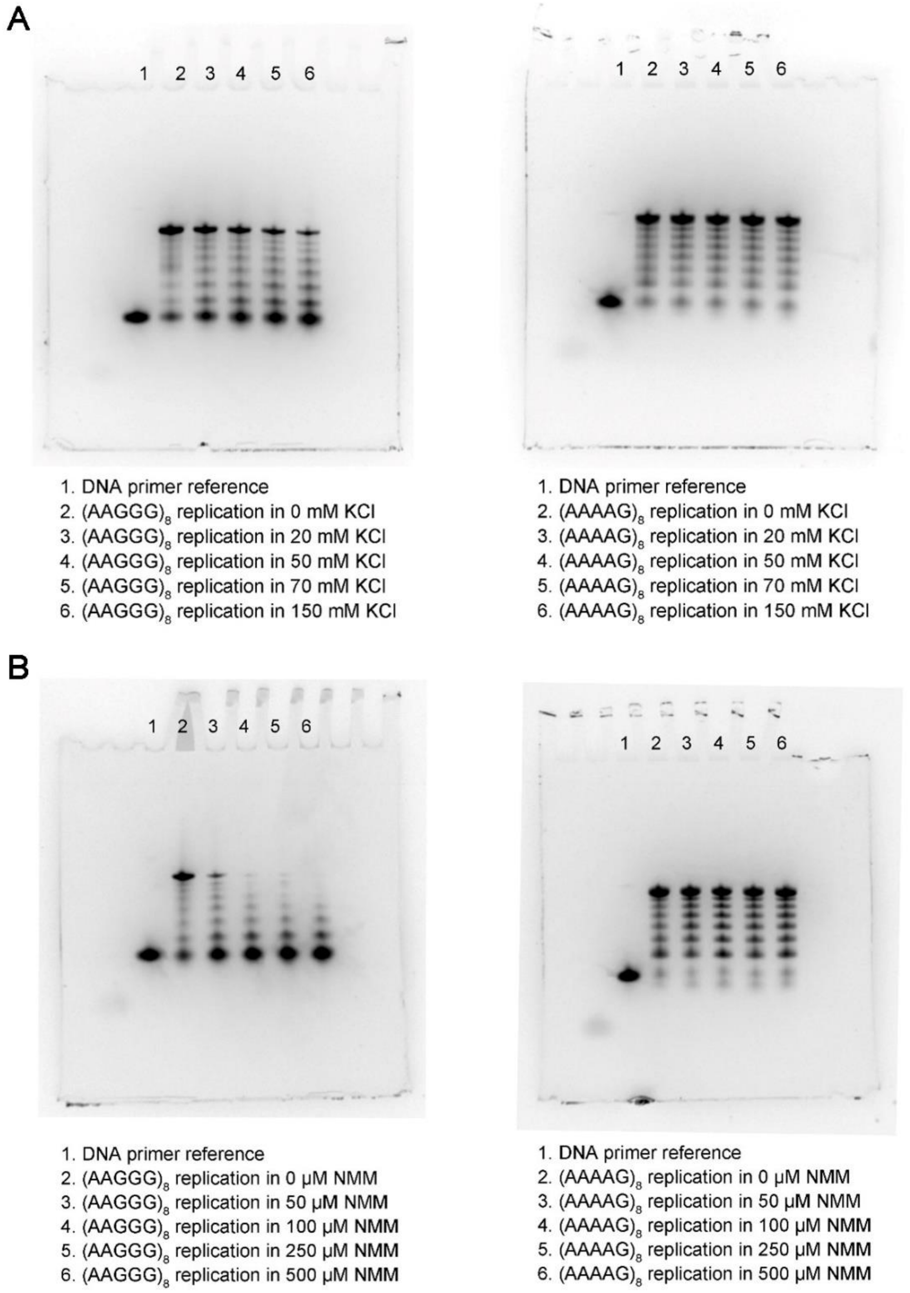

Figure S16. (A) Uncropped original gels for Figure 6B in the main text. (B) Uncropped original gels for Figure 6C in the main text.

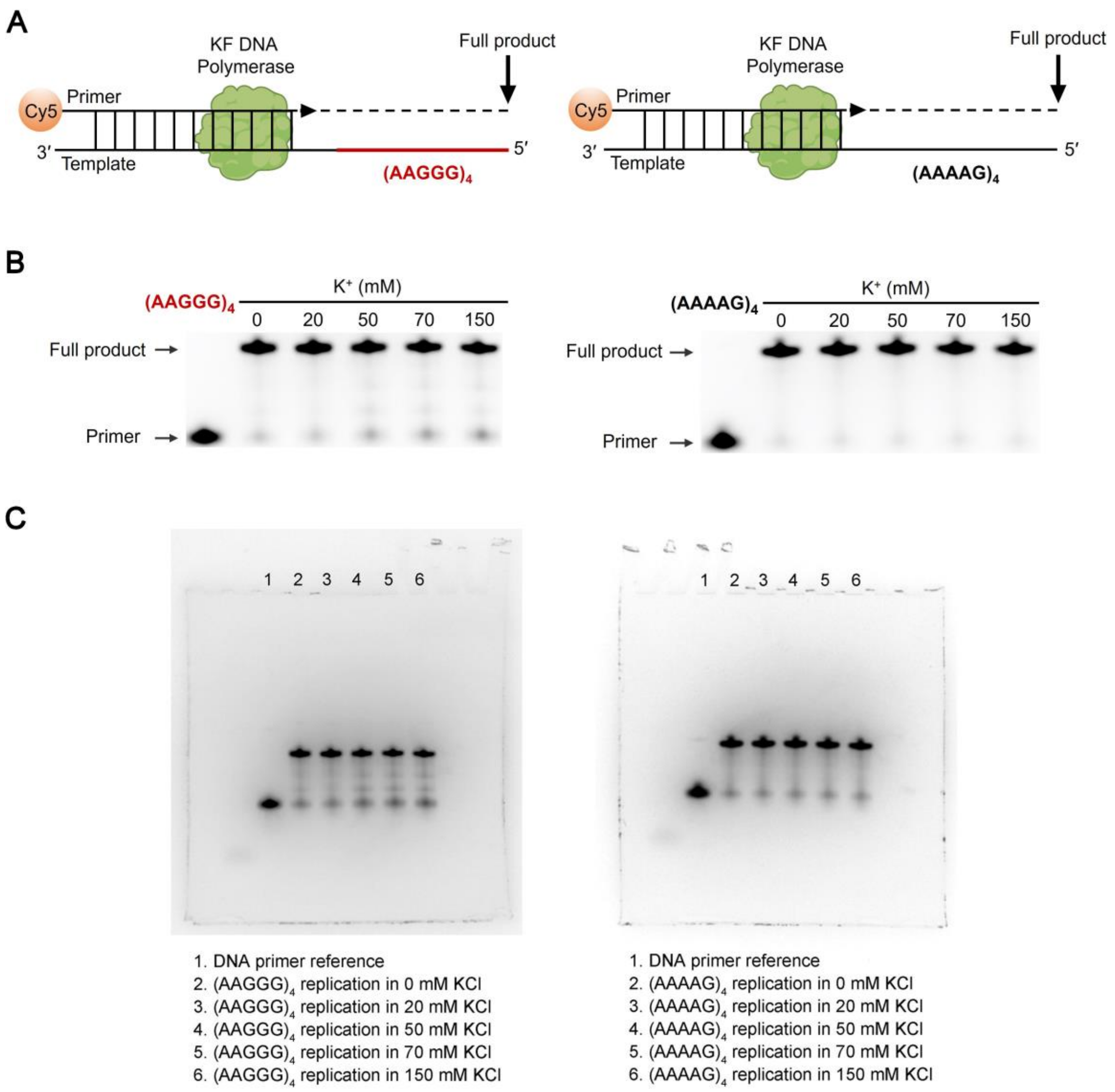

Figure S17. (A) Schematic of the *in vitro* KF extension assay on primer-template models containing four pathogenic AAGGG repeats and non-pathogenic AAAAG repeats. (B) Denaturing PAGE results show KF extension products under various concentrations of K^+. (C) The uncropped original gels for (B).

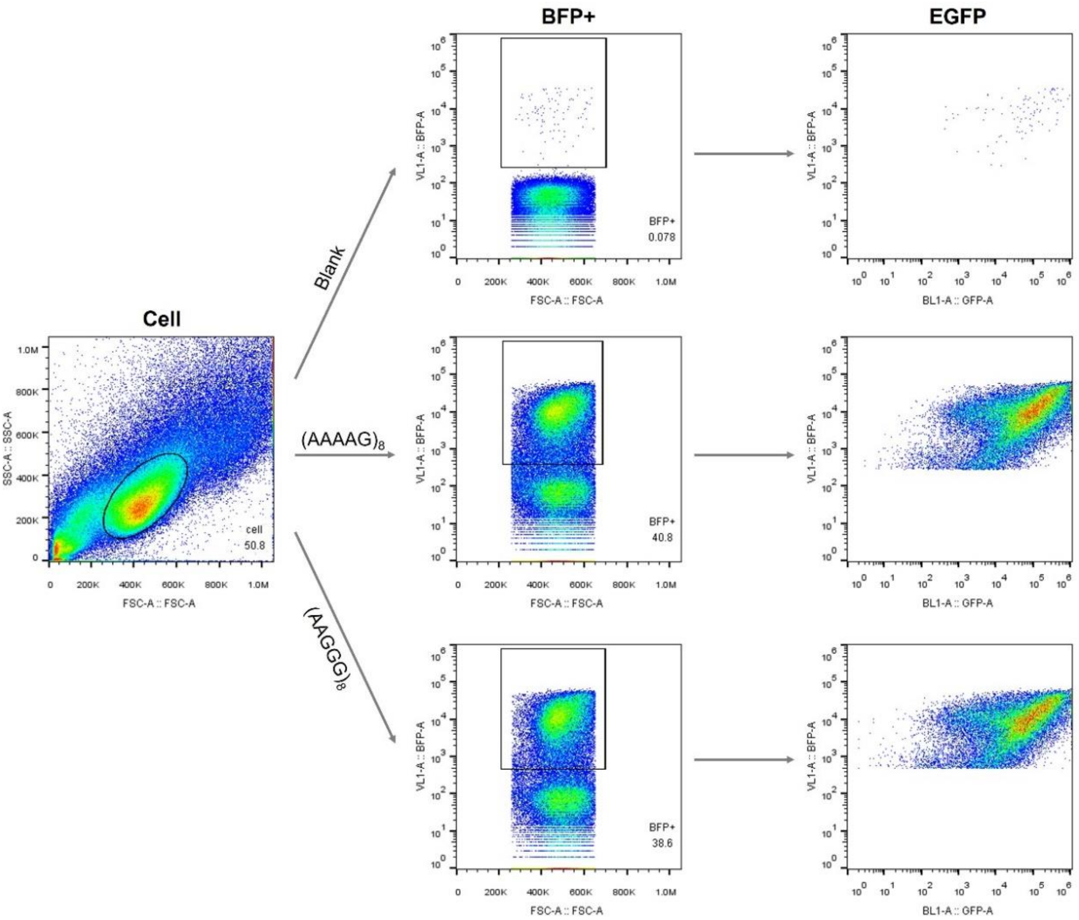

Figure S18. Representative images of the fluorescence profile of HEK293T population in flow cytometry analysis.

References

1. Phan, A.T. and Patel, D.J. (2003) Two-repeat human telomeric d(TAGGGTTAGGGT) sequence forms interconverting parallel and antiparallel G-quadruplexes in solution: distinct topologies, thermodynamic properties, and folding/unfolding kinetics. *J. Am. Chem. Soc.*, **125**, 15021-15027.
2. Mukundan, V.T., Do, N.Q. and Phan, A.T. (2011) HIV-1 integrase inhibitor T30177 forms a stacked dimeric G-quadruplex structure containing bulges. *Nucleic Acids Res.*, **39**, 8984-8991.
3. Adamczyk, B., Zurkowski, M., Szachniuk, M. and Zok, T. (2023) WebTetrado: a webserver to explore quadruplexes in nucleic acid 3D structures. *Nucleic Acids Res.*, **51**, W607-W612.
4. Guo, P. and Lam, S.L. (2016) The competing mini-dumbbell mechanism: new insights into CCTG repeat expansion. *Signal Transduct Target Ther.*, **1**, 16028.
